# Ocular neuroinflammatory response secondary to SARS-CoV-2 infection-a review

**DOI:** 10.3389/fimmu.2025.1515768

**Published:** 2025-02-04

**Authors:** Yun Zhao, Ying Tang, Qi Yao Wang, Jia Li

**Affiliations:** Department of Glaucoma, The Second Hospital of Jilin University, Changchun, China

**Keywords:** COVID-19, SARS-CoV-2, neuroinflammation, ocular complications, ocular manifestations

## Abstract

With the consistent occurrence of severe acute respiratory syndrome coronavirus 2 (SARS-CoV-2) infection, the prevalence of various ocular complications has increased over time. SARS-CoV-2 infection has been shown to have neurotropism and therefore to lead to not only peripheral inflammatory responses but also neuroinflammation. Because the receptor for SARS-CoV-2, angiotensin-converting enzyme 2 (ACE2), can be found in many intraocular tissues, coronavirus disease 2019 (COVID-19) may also contribute to persistent intraocular neuroinflammation, microcirculation dysfunction and ocular symptoms. Increased awareness of neuroinflammation and future research on interventional strategies for SARS-CoV-2 infection are important for improving long-term outcomes, reducing disease burden, and improving quality of life. Therefore, the aim of this review is to focus on SARS-CoV-2 infection and intraocular neuroinflammation and to discuss current evidence and future perspectives, especially possible connections between conditions and potential treatment strategies.

## 1 Introduction

Coronavirus disease 2019 (COVID-19), a highly contagious multisystem disease caused by severe acute respiratory syndrome coronavirus 2 (SARS-CoV-2) infection, has been reported as an unprecedented threat to the human population (1). As a global pandemic between February 2020 and May 2023, COVID-19 resulted in 776 million cases and 7.07 million deaths reported by the World Health Organization (WHO) to date, and the percentage of samples testing positive for SARS-CoV-2 is now approximately 9.85%(world, week 17 November 2024); meanwhile, the 28-day prevalence of various variants of SARS-CoV-2 ranged from 0.22% to 53.59% (2). SARS-CoV-2 has been confirmed to infect host cells predominantly through the binding of its spike (S) protein to the angiotensin-converting enzyme 2 (ACE2) receptor, which is expressed at high levels in many tissues (3–6).

Neuroinflammation, which is mediated by increases in cytokines and chemokines, reactive oxygen species (ROS) and second messenger lipids produced by astrocytes and microglia, and endothelial and peripheral immune cells (7), has been shown to significantly affect cognition and behaviour in animal models (8, 9). The persistent activation of microglia and other immune cells within the central nervous system (CNS) has a potentially damaging effect on chronic neuroinflammation (10, 11). In a few reports, this type of autoimmune response in patients with COVID-19 is considered to involve acute CNS diseases and conditions, such as encephalitis, meningitis, acute necrotizing myelitis, acute disseminated encephalomyelitis, acute ischaemic stroke, and chorea (12–19). Two years of pandemic experience have shown that approximately 13% of infected patients suffer from severe neurological complications (20). In a prospective tertiary centre cohort with a 3-month follow-up of 61 COVID-19 patients, more than forty central or peripheral nervous system complications were identified among 28 patients of them(45.9%), and the most common central nervous system complication was encephalopathy(n=19, 31.1%) (21). Some histological studies have shown that neuroinflammation, characterized by retinal glial cell proliferation and T-cell infiltration, parallels ongoing neurodegeneration in space and time (22–24). Neuroinflammation is considered an important pathogenic factor of neurodegenerative lesions (25).

Some patients have shown ocular symptoms as their primary symptoms or developed ocular complications, and there has been a significant increasing trend in the number of patients with ophthalmic diseases associated with SARS-CoV-2 infection. Approximately 11% of patients develop ocular disease after SARS-CoV-2 infection, and an average of 13.6% of patients with COVID-19 develop follicular conjunctivitis (26). Conjunctivitis is considered the main manifestation among the current reports, but other inflammatory reactive ocular diseases and conditions, such as optic neuritis, ocular vascular diseases, glaucoma, orbital neuropathy, xerophthalmia and muscae volitantes, have also been reported (27–42). A study revealed that patients who had COVID-19 were at greater risk (hazard ratio 1.18, 95% CI 1.03–1.34) of developing a new diagnosis of uveitis than were matched controls within multiple time periods between one-month and two-years (43). Among the 117 patients with severe COVID-19 examined, forty-two patients had ophthalmological manifestations (unilateral in 23 patients and bilateral in 19 patients), 10 of these patients had more than one ophthalmological manifestation, and the most frequent fundoscopic findings were optic nerve inflammation, microvasculature occlusion, and major vascular occlusions (44).

It has been widely confirmed that SARS-CoV-2 infection can induce an immunological response, followed by unique changes in the numbers of immune cells and the levels of chemokines, cytokines and inflammatory molecules, as well as some neurological symptoms (45, 46). Persistent inflammation, as evidenced by elevated levels of some inflammation-related factors within ocular tissue secondary to SARS-CoV-2 invasion, appears to contribute to long-standing ocular symptoms.

To date, although prior studies have suggested potential mechanisms underlying neuroinflammation secondary to COVID-19, reports on postinfection ocular manifestations and their associations with intraocular neuroinflammation are limited. However, these conditions present a challenge for ophthalmologists and researchers. Further investigations are needed to identify the persistent ocular effects after SARS-CoV-2 infection. Within this context, we analysed the associations among SARS-CoV-2 infection, ocular complications and intraocular neuroinflammation.

## 2 Ocular manifestations and neuroinflammatory response of COVID-19

The fact that ocular manifestations can be the first presenting feature of COVID-19 should not be ignored (27). To our knowledge, the ocular manifestations of COVID-19 mainly involve the conjunctiva, cornea, sclera, anterior chamber, pupils, retina, optic nerve and visual cortex, extraocular muscles and their innervating cranial nerves, orbit and lacrimal system (47). Ocular complications have been reported in some patients with COVID-19 (**Table 1**). In addition, diplopia and blepharoptosis can also be found in patients with COVID-19 (48–51).

**Table 1:** Common ocular complications reported in SARS-CoV-2-infected individuals.

Country	Age, years	Gender ratio, M/F	N/T or ORs (T)	Ocular complications	Author
Brazil	62.50	9/9	55.56% (18)	Retinal flame-shaped haemorrhages Ischaemic pattern lesions (cotton wool spots and retinal sectorial pallor)	Leonardo Amarante Pereira et al. (135)
China	68.00	25/13	31.58%(38)	Conjunctival congestion Tear overflow Conjunctival oedema	Ping Wu et al. (34)
China	48.00 ± 12.10	31/25	26.79%(56)	Sore eyes Itching Foreign body sensation Tearing Redness Dry eyes Eye secretions Floaters	Nan Hong et al. (37)
China	58.68 ± 14.81	36/36	2.78%(72)	Conjunctivitis	Xian Zhang et al. (136)
China	57.50	Male prevalent	1.10%(1167)	Conjunctivitis	Lorenzo Loffredo et al. (137)
China	44.00	253/255	5.05%(535)	Blurry vision Conjunctival congestion Increased conjunctival secretion Ocular pain Photophobia Xerophthalmia Tearing	Liwen Chen et al. (138)
China	47.00	459/637	0.82%(1096)	Conjunctival congestion	Wei-Jie Guan et al. (139)
China	54.50 ± 14.17	21/9	3.33%(30)	Conjunctivitis	Jianhua Xia et al. (140)
France	40.70 ± 20.30	197/303	64.20% (500)	Pterygium Diplopia Hordeolum Corneal abscess Recurrent corneal erosion Optic neuritis Macular disorder Angle-closure Vitreoretinal disorder Blepharitis Uveitis Conjunctivitis Foreign body sensation Subconjunctival hemorrhage	H. Bourdon et al. (141)
Germany	37.90 ± 13.70	51/57	69.40% (108)	Burning sensations Itching Epiphora (watering eyes) Mucoid discharge Purulent discharge Photophobia Foreign body sensation Conjunctival swelling Eyelid swelling Feeling of pressure Double images Metamorphopsia Redness Reduced visual acuity Pain	Alexander C. Rokohl et al. (142)
India	38.80	113/14	6.29% (127)	Conjunctivitis	K Sindhuja et al. (143)
Iran	32.60 ± 15.00	77/65	64.50% (142)	Conjunctival hyperemia Keratitis Cataract Diabetic retinopathy Epiphora Hyperemia Eye irritation Itching sensation Foreign body sensation Ocular pain Photophobia Blurred vision	Mojtaba Abrishami et al. (144)
Italy	7.00	20/7	14.84%(27)	Mild viral conjunctivitis	Paola Valente et al. (145)
Italy	77.10 ± 12.60	15/14	34.48%(29)	Eye burning Foreign body sensation Tearing Conjunctival hyperaemia and/or chemosis Lid margin hyperaemia and/or telangiectasia Crusted eyelashes Meibomian orifices alterations	Alessandro Meduri et al. (146)
Italy	49.90 ± 15.60	38/16	44.35%(54)	Retinal haemorrhages Cotton wools spots Dilated veins Tortuous vessels	Alessandro Invernizzi et al. (147)
Italy	70.00	25/18	7.00% (43)	Bilateral conjunctivitis Unilateral posterior chorioretinitis of opportunistic origin	Maria Pia Pirraglia et al. (148)
Korea	FG: 49.00 ± 18.00 RG: 44.00 ± 16.00	FG: 15/56 RG: 8/24	T: 21.36% (103) FG: 16.90% (71) RG: 31.25% (32)	Conjunctival congestion Ocular discomfort Ocular pain Visual disturbance Epiphora Itching sensation	You Hyun Lee et al. (149)
Spain	56.00	18/9	22.00%(27)	Retinal cotton wool exudates	M F Landecho et al. (150)
Turkey	58.48 ± 15.89	197/162	4.50%(359)	Conjunctivitis Subconjunctival hemorrhage Vitreous hemorrhage Conjunctival hyperemia Epiphora Eye secretion Photophobia	Hasan Öncül et al. (151)

*Age, years: Median/Mean ± SD;

*FG, First-episode group;

*RG, Relapsed group;

*M/F, Male/Female;

*N, The number of included subjects with ocular complications following SARS-CoV-2 infection in the reported study, observation or meta-analysis;

*T, The number of all included subjects with SARS-CoV-2 infection in the reported study, observation or meta-analysis;

*ORs, Odds ratios in the meta-analysis.

Previous studies have indicated that SARS-CoV-2 has ocular tropism and can infect the eyes directly or migrate from the respiratory tract to the brain and eye via the trigeminal and optic nerves and survive (45). Similar to the blood-brain barrier(BBB), the blood-retinal barrier(BRB), which consists of the outer barrier of the RPE and the inner barrier of the retinal vascular endothelium, is an important component of ocular health, regulating metabolic homeostasis while maintaining the immune privilege. Notably, the study of Monu Monu et al. provided the first evidence of SARS-CoV-2 ocular tropism via cells lining the BRB and that the virus can infect the retina via systemic permeation and induce retinal inflammation (52). This is comparable to the Ebola virus, which disrupts the BRB, leading to ocular complications. Ebola virus-like particles stimulate pericytes to secrete VEGF, leading to rupture of the inner BRB, but do not cause direct cytotoxic effects on retinal endothelial cells (53). SARS-CoV-2 is known to invade human cells through engagement with specific membrane cell receptors, which include the ACE2 transmembrane receptor and activation of the SARS-CoV-2 S protein by transmembrane serine protease 2 (TMPRSS2) cleavage (6, 54–56). CD147 plays an important role in facilitating SARS-CoV-2 invasion of host cells, and CD147 is expressed in tear fluid, aqueous humour and the vitreous humour (57). In addition, ACE2 has been shown to be expressed in the human conjunctiva, cornea, aqueous humour and retinal cells (58–60). These findings suggest that ocular tissues may also be target tissues for SARS-CoV-2 infection. In an animal model, SARS-CoV-2 infection promoted retinal inflammation, and immunofluorescence analysis of ocular tissues from infected mice suggested that intraocular T-cell and neutrophil counts were significantly elevated, whereas the production levels of proinflammatory cytokines and the chemokines G-CSF, IP-10, MCP-1, MIP-2, IL-6 and IL-2 were increased (61).

Through autopsies of eye tissues of COVID-19 patients, the study of H Nida Sen et al. have reported the localization of SARS-CoV-2 RNA in the ocular tissues demonstrated by *in situ* hybridization, including neuronal cells of the retinal inner and outer layers, ganglion cells, corneal epithelia, scleral fibroblasts and oligodendrocytes of the optic nerve (62). SARS-CoV-2 RNA and SARS-CoV-2 S and nucleocapsid (N) proteins were detected in the eyes of patients who had recovered from COVID-19 (27, 28, 41), whereas IFN-γ, TNF-α, IL-5, IL-8, and granulocyte−macrophage colony−stimulating factor (GM−CSF) expression levels were significantly elevated in the tear film of patients with SARS-CoV-2 RNA-positive conjunctival swabs (27). Cytokine expression levels in the eyes may be independent of plasma cytokine expression levels. Previous studies have shown greater expression levels of cytokines such as IL-7, IL-8, IL-10, IFN-γ, G-CSF, interferon-inducible protein 10 (IP-10) and IL-1α in the plasma of patients with cytomegalovirus retinitis (CMVR) than in those of patients with ocular syphilis, but in aqueous humour, the levels of IL-1α, IP-10 and GM-CSF were greater (63). Therefore, the immunological characteristics of the aqueous humour and plasma are independent of each other, and the cytokine levels in the aqueous humour cannot be inferred from the cytokine levels in the plasma of patients.

However, previous studies focused mostly on changes in intraocular cytokine expression after SARS-CoV-2 infection in animal models or autopsies. Therefore, further *in vivo* studies of human samples are needed.

### 2.1 Ocular surface diseases and manifestations associated with COVID-19

Ocular surface complications often occur in patients with SARS-CoV-2 infection (64). For example, five cases of nonremitting conjunctivitis as the sole presenting sign and symptom of COVID-19 have been reported; these patients tested positive for SARS-CoV-2 infection via RT−PCR of nasopharyngeal swabs but developed no fever, malaise or respiratory symptoms throughout their infection period (65). In another report, a patient with COVID-19 initially presented with keratoconjunctivitis, with respiratory symptoms appearing four days later (66). Similarly, episcleritis has also been described as a possible presenting sign of COVID-19 (67).

A random-effects meta-analysis of 8219 patients with COVID-19 revealed an estimated prevalence of ocular manifestations of 11.03% (95% confidence interval [CI]: 5.71–17.72), and the most common ocular manifestations were dry eye or foreign body sensation (16%), redness (13.3%), tearing (12.8%), itching (12.6%), eye pain (9.6%) and discharge (8.8%) (68). Considering studies of certain children hospitalized with COVID-19, ocular surface manifestations, such as conjunctival discharge, eye rubbing and conjunctival congestion, were associated with systemic clinical symptoms or cough (69). The ocular surface symptoms eventually recovered or were ameliorated (69). Another meta-analysis involving 5717 patients with COVID-19 revealed ocular manifestations, including conjunctival hyperaemia (7.6%), conjunctival discharge (4.8%), epiphora (6.9%) and foreign body sensation (6.9%) (70). In addition, the positive rate of conjunctival swab tests was 3.9%, and severe cases of COVID-19 were associated with an increased risk of developing ocular complications (odds ratio [OR]=2.77, 95% CI 1.75–4.40) (70).

Viral conjunctivitis is the most common ocular manifestation of COVID-19, generally with symptoms such as conjunctival congestion, pain, foreign body sensation and increased secretion (40, 47). Moreover, alterations in ocular protection mechanisms promote the extreme susceptibility of the corneal surface to exposure keratopathy and microbial keratitis (64, 71). Patients with COVID-19 have been reported to have an increased risk of microbial keratitis with prolonged hospitalization in the intensive care unit (72, 73). The ocular surface serves not only as a direct connection between the eye and the outside environment but also as a connection to the respiratory tract through the nasolacrimal duct and nasal cavity. The positive viral nucleic acid test results confirmed in conjunctival sac swabs and tear samples from patients with COVID-19 combined with conjunctivitis provided objective evidence of SARS-CoV-2 infection on the ocular surface (27, 28, 30, 41, 74, 75).

However, persistent dry eye disease induces major central neuroinflammatory responses known to participate in chronic pain, and such central neuroinflammatory responses may participate in the development of the chronic ocular pain observed in patients with dry eye disease (76, 77). Several studies have shown that females and older patients are at increased risk of developing long COVID-19 (45, 78–82). Additionally, female sex and older age are associated with neurological manifestations of long COVID-19 (83). Female sex and older age are also susceptibility factors for some ocular diseases, such as xerophthalmia (84–86).

Additionally, patients with ocular surface symptoms (such as epiphora, conjunctival congestion or chemosis) are more likely to have higher white blood cell counts; neutrophil counts; and procalcitonin, C-reactive protein and lactate dehydrogenase levels in their blood test results than patients without these manifestations (34). Presumably, SARS-CoV-2 invasion of ocular cells and subsequent long-lasting local inflammation may lead to noticeable changes in these inflammatory indicators, which may be drivers of persistent ocular manifestations.

### 2.2 Ocular anterior segment diseases and neuroinflammatory response associated with COVID-19

Anterior segment changes, such as acute atrial angle closure (64) and bilateral acute iris transillumination (87–90), have also been reported to occur after SARS-CoV-2 infection. The pathogenesis of ocular anterior segment diseases associated with SARS-CoV-2 infection needs to be further investigated but may be directly or indirectly induced by viral infection; alternatively, ocular complications may develop during the treatment of systemic symptoms of COVID-19.

Acute uveitis, including acute blurry vision, photophobia, redness, ocular hypertension, pain and iris atrophy discolouration, has been observed in many patients following SARS-CoV-2 infection (41, 87, 88, 90, 91). Evidence suggests that cough, sore throat, hyponatremia, prone positioning and high-dose steroid therapy can be triggers for ocular diseases secondary to COVID-19 (64, 92).

According to a few reports, several patients develop an acute attack of glaucoma after contracting COVID-19 (28, 41, 91). Patients with COVID-19 often present with symptoms such as fever, cough and sore throat, and the incidence of negative manifestations such as insomnia, anxiety and depression has also increased compared with that in the preepidemic period (93, 94). All these factors may contribute to elevated intraocular pressure. In addition, the treatment of patients with COVID-19 with high-dose glucocorticoid therapy, the consumption of large amounts of water and the use of some cough and antipyretic drugs are potential factors for inducing acute attacks in high-risk groups with local ocular anatomical features of closed-angle glaucoma. Sedat Özmen et al. reported the cases of three patients with COVID-19 and hyponatremia who also had acute angle-closure glaucoma (AACG) (95). Another case of AACG was shown to have occurred in a patient with severe COVID-19 treated with prone position ventilation (96). Guido Barosco et al. described a case in which bilateral primary angle closure (PAC) progressed to unilateral end-stage primary angle closure glaucoma (PACG) associated with treatment for COVID-19. The patient’s severe clinical condition and prolonged systemic therapy masked the symptoms and delayed the diagnosis, suggesting that COVID-19 treatment may pose an increased risk for PAC (97). The use of antiviral drugs has also been correlated with glaucoma. A study of a cohort of Australian individuals revealed an increased incidence of glaucoma medication usage in middle-aged Australian males taking antiretroviral medication (98). A few clinical case studies have revealed the development of bilateral AACG with the use of oseltamivir (99, 100). Although there is no clear evidence that antiviral drugs increase the incidence of glaucoma, it is reasonable to speculate that this may be related to drug-induced toxicity, high intraocular pressure or an idiosyncratic drug response (98).

In recent years, many studies have suggested that, similar to other neurodegenerative diseases of the CNS (such as Alzheimer’s disease, PD, and multiple sclerosis), glaucoma can be considered an autoimmune disease mediated by autoreactive T cells, and all of these diseases are characterized by impaired barrier function and chronic neuroinflammation triggered by autoantigens (101, 102). The level of ACE in the aqueous humour of patients with glaucoma is significantly greater than that in nonglaucoma patients; furthermore, the expression of ACE in the aqueous humour of patients without glaucoma is lower than that of ACE2, which controls intraocular pressure by interacting with ACE (103). Experiments in animals revealed that the hypotensive effect of ACE2 works through the ACE2–Ang(1-7)–MasR axis and that the activation of ACE2 contributes to decreasing the expression of caspase-3 and reducing the death of RGCs, thus protecting nerve fibres and RGCs (104). Furthermore, SARS-CoV-2 infection has been shown to decrease ACE2 expression in lung tissue and lead to the generation of proinflammatory cytokines such as IL-6 (105–107). Overall, the activation of endogenous ACE2 could be a strategy for the treatment of optic nerve damage.

Furthermore, intraocular neuroinflammation is most likely associated with the immune responses of T cells in patients with COVID-19. Increased TGF-β1 and TGF-β2 expression levels in aqueous humour during acute episodes of AACG have been reported in studies of individuals from the Asia–Pacific region, and cytokine levels are closely related to temporary ischaemic damage to the eyes (108). The dynamic balance of cytokines and chemokines is essential for maintaining intraocular homeostasis, and their changes also reflect the metabolic and immune status of intraocular tissues. IgG and plasma cells have been shown to be deposited in the retinas of patients with glaucoma and can maintain the proinflammatory environment via microglia (109). Moreover, in glaucoma, the activation of immunoreactive cells (microglia and macroglia) within the retina and the infiltration of peripheral immune cells (e.g., T cells, B cells, macrophages, and monocytes) are connected with the apoptosis of RGCs (109–111).

Some reports have shown that SARS-CoV-2 infection can cause secondary glaucoma (112, 113). Many studies have demonstrated the permissivity of the trabecular meshwork towards viruses with a concomitant increase in IOP in animal models (113). Virus-induced uveitis disrupts the blood–aqueous humour barrier, facilitating the entry of inflammatory cytokines and immune cells into the intraocular environment (114). Manoj Soman et al. reported the case of a male patient who developed neovascular glaucoma (NVG) 5 weeks after SARS-CoV-2 infection, suggesting that the COVID-19-associated prothrombotic state secondary to retinal vascular involvement has the potential to trigger this type of glaucoma (115).

Considering the ocular tropism of SARS-CoV-2 and the similar pathophysiologic mechanisms of glaucoma and uveitis in terms of oxidative stress, immune response-mediated inflammation, it is possible that SARS-CoV-2 infection can induce AACG and uveitis. Studies by Ron NeumannPrivate and Alejandra de-la-Torre et al. demonstrated that patients with herpes simplex virus or cytomegalovirus-infected uveitis presented with elevated intraocular pressure (IOP), iris atrophy and pupil dilation (116, 117). Some populations are susceptible to AACG because of their inherently dangerous anatomy and may need to experience a long period of time to develop in the absence of stressors. SARS-CoV-2 infection may be an intense stressor that accelerates the progression. Yue Ying et al. compared 171 patients with acute primary angle-closure (APAC) and found that there was a surge in the incidence of COVID-19-positive group in the Asia-Pacific region compared to the COVID-19-negative group on December 22, 2022, which coincided with an increase in the COVID-19 antigen-positive population, and that COVID-19-positive patients with APAC were younger, with a significantly greater depth of the anterior chamber, and with pupil dilation was higher (118).

### 2.3 Ocular posterior segment diseases and neuroinflammatory response associated with COVID-19

Ocular diseases of the posterior segment, such as optic neuritis, retinal vascular occlusion, ischaemic optic neuropathy and orbital osteofascial compartment syndrome, are not infrequent among SARS-CoV-2 infection-associated ophthalmopathies (64, 119).

Optic nerve damage in patients with COVID-19 is characterized by subacute visual loss, visual field defects, relative afferent pupillary block, pain with movement, optic disk oedema and optic nerve thickening (47). Furthermore, SARS-CoV-2 infection causes widespread inflammation and cerebral venous thrombosis, which lead to intracranial pressure, decreased vision and optic disk oedema (120). Bosello F et al. described a patient who developed myelin oligodendrocyte glycoprotein (MOG) antibody-associated unilateral retrobulbar optic neuritis a few weeks after asymptomatic COVID-19 and subsequently experienced acute inflammatory demyelinating polyneuropathy after the resolution of optic neuritis (121).

Central retinal vein occlusion (CRVO) is a common vitreoretinal disease reported in patients with COVID-19 (33). Several cases of CRVO secondary to COVID-19 with visual loss, fundus macular oedema and retinal haemorrhage have been reported (122–124). Retinal circulation dysfunction should be considered a potential aetiology of ocular manifestations in patients with COVID-19. Several parallels, such as anterior ischaemic optic neuropathy and choroidal ischaemia, have been described in patients with COVID-19 (125). There was a significantly greater likelihood of retinal microangiopathy in 972 patients with COVID-19 than in controls; moreover, optical coherence tomography angiography (OCTA) revealed reduced vessel density and an enlarged foveal avascular zone in patients with COVID-19 (126). Compared with controls, holistic analysis of retinal markers revealed that the retinal microvasculature changed, inflammation increased and gliosis occurred in eyes afflicted by COVID-19; concurrently, analysis of the choroidal vasculature revealed localized changes in density and signs of increased inflammation in COVID-19 samples (127).

Many studies have suggested that COVID-19-related retinal microangiopathy is a significant ocular manifestation of COVID-19 that can be used to predict future retinal complications. In addition, these microvascular impairments regularly occur before clinically visible changes occur, and OCTA may be used for early detection.

It has been reported that functionally active autoantibodies against G protein-coupled receptors (GPCR-AAbs) are observed in patients after SARS-CoV-2 infection, and a study revealed that GPCR-Aab seropositivity can be linked to impaired retinal capillary microcirculation, potentially mirroring the systemic microcirculation with consecutive clinical symptoms (128). Another study revealed that neutralization of GPCR-AAbs can improve retinal capillary microcirculation in patients with glaucoma who have recovered from COVID-19 (129).

H Nida Sen et al. have found six eyes showed potential COVID-19-associated macroscopic lesions in the retina through postmortem human eyes from 25 patients with confirmed SARS-CoV-2 infection, the retinal lesions potentially associated with SARS-CoV-2 infection included small neovascularization, retinal sclerotic vessels, retinal vascular occlusion with fibrin/thrombi in retinal vessels and vitreous hemorrhages (62).

Owing to the damaging effects of neuroinflammation and viral invasion of the optic nerve, an abnormal neurological immunological response develops in the intraocular environment. Although this neuroinflammatory state has not been revealed to directly result in ocular manifestations, it may contribute to disease progression through chronic activation of specific inflammatory molecules and immune cells and dysfunction of the BRB, whereas immunocyte activation can promote excessive release of inflammatory mediators. With ongoing cytokine abnormalities and autoantibody generation, specific inflammatory molecules and immune cells can be chronically active, leading to impaired BRB integrity and neurodegeneration. In addition to autoantibody generation, this persistent inflammatory response results in T-cell dysregulation, which is also related to BRB dysfunction and microcirculation disorders.

### 2.4 Other ocular diseases and neuroinflammatory response associated with COVID-19

Because the ACE2 receptor can be detected in many intraocular tissues, through SARS-CoV-2 infection, COVID-19 may contribute to persistent intraocular neuroinflammation, microcirculation dysfunction, ischaemic neuronal injury and ocular symptoms in addition to known multisystem disturbances.

During the pandemic, facial nerve palsy, ocular movement abnormalities and vestibular alterations were reported in the paediatric population (130, 131); moreover, in children, cases of acute acquired concomitant esotropia not caused primarily by neuritis were reported (132, 133).

Opsoclonus–myoclonus–ataxia syndrome (OMAS) is a rare neurological syndrome characterized by saccadomania or spontaneous conjugate multidirectional eye movements, myoclonus in the limbs, and ataxia (134). A 57-year-old man who presented with OMAS was positive for SARS-CoV-2 infection according to a PCR test; this is the first reported case of OMAS associated with COVID-19 (134). Darion L Heald et al. reported a case of opsoclonus after COVID-19 in an infant (14).

Taken together, these findings suggest that COVID-19 eye disease can be the first symptom, complication or the only manifestation in patients with SARS-CoV-2 infection. Differences in study participants, such as race, age, past medical history, chronic underlying medical conditions, and severity of COVID-19, could be the reason for the distinct incidence of various ocular complications across different cases or analyses.

### 2.5 Ocular adverse effects of COVID-19 vaccination

Similar to the ocular manifestations of COVID-19. The Ocular adverse effects of COVID-19 vaccines include facial nerve palsy, abducens nerve palsy, acute macular neuroretinopathy, central serous retinopathy, thrombosis, uveitis, multiple evanescent white dot syndrome, Vogt-Koyanagi-Harada disease reactivation, and new-onset Graves’ disease (152).

A retrospective chart review among 25 patients(76% females, mean age 43.2 years) revealed that anterior uveitis was the most common type of uveitis (56%), 19 patients(76%) developed uveitis after COVID-19 vaccination, and 15.8% of patients needed increased systemic therapy during post-vaccine uveitis (153). The primary analysis of Zhenyu Zhong et al. included 438 non-COVID-19 participants(median age 41 years and 57.3% female), with 857 doses of COVID-19 vaccination in total, this study revealed that a total of 39 episodes of uveitis relapse events occurred in 34 patients after the receipt of a dose of COVID-19 vaccine within 30 days, and concomitant use of systemic glucocorticoids at the time of vaccination was independently associated with a decrease in the risk of relapse after vaccination (HR, 0.23 [95% CI, 0.07-0.74]; P value = 0.014) (154). Another retrospective study from December 11, 2020, to May 9, 2022, reported a total of 1094 cases of vaccine-associated uveitis (VAU) in 40 countries, with estimated crude reporting rates (per million doses) of 0.57, 0.44 and 0.35 for BNT162b2, mRNA-1273 and Ad26.COV2.S respectively, the mean age of patients with VAU was 46.24 ± 16.93 years, and 68.65% (n=751) were females; most cases occurred after the first dose (n=452, 41.32%) and within the first week (n=591, 54.02%) of vaccination (155).

As the rate of COVID-19 vaccination has gradually increased, related adverse events, including optic neuritis, have been reported. Christian García-Estrada et al. reported the case of a woman with unilateral optic neuritis one week after COVID-19 vaccination (156). W-J A Lee described a similar case of a woman with unilateral optic neuritis one week after COVID-19 vaccination (157). Hiroyasu Katayama et al. reported the case of a healthy man who was diagnosed with bilateral optic neuritis after COVID-19 vaccination (158). Khalid Sawalha et al. reported a case that elucidated an interesting and rare finding for the presentation of COVID-19 with optic neuritis, and this patient also suffered from myelin oligodendrocyte glycoprotein-IgG-associated disease (MOGAD) (159). Ayman G. Elnahry et al. reported the cases of two women who developed optic neuropathy after COVID-19 vaccination, with clinical manifestations of optic papillary oedema and progressive blurred vision (119), suggesting that neuroimaging and cerebrospinal fluid analysis may be helpful in determining the cause of vision loss in these patients. Although a clear connection cannot be confirmed because of insufficient evidence, these cases may help guide further research on the potential pathogenesis of optic neuritis related to SARS-CoV-2 infection.

Several case reports have shown that abnormal hypercoagulability and increased risk of thromboembolism are not uncommon during pandemics. Among the 26 patients who were vaccinated with either mRNA or adenoviral vector vaccines for COVID-19 and presened with retinal vascular occlusions, there were more retinal vein occlusions than retinal artery occlusions, and ocular symptoms mostly occurred within 3 weeks after vaccination (160).

Studies have shown that compared with the influenza vaccine, the COVID-19 vaccine has a greater relative risk of allergies, general cardiovascular events, coagulation, bleeding, ocular adverse effects(AstraZeneca RR39.28; Pfizer-BioNTech RR34.51), and especially thrombosis, compared to the influenza vaccine (161). There are more than 20 described cases of new-onset uveitis after vaccination with the ChAdOx1 nCoV-19 (Oxford/AstraZeneca) and BNT162b2 (Pfizer/BioNTech) vaccines, with the onset of symptoms varying from one to 30 days (162). Peixuan Zhang et al. reported a total of 9 cases of IgG4-related ophthalmic disease, of which 5 developed symptoms after COVID-19 vaccination and 4 developed symptoms after SARS-CoV-2 infection (163).

In a self-controlled case series study, COVID-19 vaccines were administered to 81 people, 25 (31%) of whom developed thyroid eye disease(TED) in the exposed period and 56 (69%) in the unexposed period, the results of the study revealed that there was an increased risk of TED after COVID-19 vaccination. In parallel, there were no differences in the clinical manifestations of TED in terms of disease duration(P=0.80), activity(P=0.48), severity(P=0.23), and treatment(P=0.80) in relation to COVID-19 vaccination status (164). Similar reports are available. A multicentre retrospective study suggested that COVID-19 vaccination may enhance the risk of non-arteritic anterior ischemic optic neuropathy (NAION); nevertheless, the overall clinical characteristics and follow-up visual condition of NAION patients vaccinated with COVID-19 were similar to those of typical NAION patients (165).

There is evidence that some retinal pigment epithelium surface proteins (MRP-4, MRP-5, RFC1, SNAT7, TAUT and MATE) can cross-react with the SARS-CoV-2 E protein and also induce autoimmune reactions after vaccination (166), which may be one of the mechanisms underlying the ocular manifestations of COVID-19 or the occurrence of ocular adverse reactions after vaccination. However, owing to the use of only a few retrospective studies or independent case reports alone, it is not possible to clarify the causality of the occurrence of ocular adverse reactions after COVID-19 vaccination, and determining each potential ocular effect of vaccination is a formidable challenge; however, it is only possible to hypothesize that it is related to the immune response to vaccination.

### 2.6 Ocular diseases and manifestations during post-COVID-19 or Long COVID

Persistent or late-onset neurological and neuropsychiatric symptoms affect a substantial fraction of people after COVID-19 and represent a major component of the post-acute COVID-19 syndrome, also known as long COVID (167). Ocular manifestations are similar. In a retrospective study of 12 COVID-19 patients without known risk factors, the median time from COVID-19 diagnosis to ophthalmic symptoms was 6.9 weeks (168). It can be hypothesized that the persistent body hypercoagulability and the inflammatory response due to SARS-CoV-2 infection may lead to an increased risk of ocular manifestations.

Notably, the cornea was susceptible to SARS-CoV-2 in the presence of SARS-CoV-2 receptors (CD147 and ACE2) and spike protein remnants (4 out of 58) in post-recovery corneal lenticules, SARS-CoV-2 infection triggered immune responses in the corneal stroma in recovered COVID-19 patients, with elevated IL-6 levels observed between 45 and 75 days post-recovery, which were then lower at approximately day 105 (169).

Yuzhu Gao et al. reported alterations in parameters of the foveal avascular zone (all p < 0.05) and hyper-reflective dots in the vitreous of 27 patients (54 eyes) (71.1% vs. pre-COVID-19, 34.2%, p=0.006) via swept-source optical coherence tomography and angiography (170). Compared with the pre-COVID-19 status, patients with 1- and 3-month post-COVID-19 statuses presented significant thinning of ganglion cells and the inner plexiform layer, thickening of the inner nuclear layer, a decrease in the vessel density of the superficial vascular complex, and an increase in the vessel density of the deep vascular complex (170).

Longer observations of ocular manifestations during post-COVID-19 have not been reported. Further longitudinal studies are needed to examine long-term changes during post-COVID-19 or Long COVID in the human eye.

## 3 Viral infection and neuroinflammation

### 3.1 Other viral infections and neuroinflammation

The predominant types of neurotropic viruses that pose a threat to human populations include herpesvirus, Epstein–Barr virus (EBV), SARS coronavirus, neurotropic enteric viruses, flaviviruses, alphaviruses and togaviruses, and a wide array of neurological manifestations can occur (171, 172). Infection of the cells in the nervous system is an integral part of the survival strategy of neurotropic viruses. Virus reactivation contributes to neuroinflammation because of the involvement of the nervous system. To our knowledge, the immune response to EBV reactivation has been shown to reflect that of myalgic encephalomyelitis (ME) or chronic fatigue syndrome (CFS) (167, 173). EBV viremia is clearly a risk factor for the development of long COVID-19 symptoms (174).

Studies in animals have demonstrated that coronavirus infections lead to retinal damage, which is associated with ocular microvascular disease, retinitis, retinal degeneration and BRB disruption (175–177). Some studies have shown that coronavirus species can also cause serious ocular diseases and conditions, including conjunctivitis, anterior uveitis, retinitis, and optic neuritis, in animal models (29). In addition, it is suggested that viral infection increases the risk of secondary bacterial infection.

Common aetiologies of viral anterior uveitis (VAU) include herpes simplex, varicella-zoster, cytomegalovirus and rubella virus (178). Additionally, EBV and Varicella-zoster virus (VZV) can remain latent in host cells after primary infection until the emergence of a stressor, such as another acute viral infection, leading to the reactivation of these herpes viruses and the induction of inflammation and neurological symptoms (45).

Enteric viruses can be transported axonally, along the enteric nervous system, through the vagus nerve and prevertebral ganglion to connect with the central nervous system, and also via the fluidic system through the BBB and blood-cerebrospinal fluid barrier (179). The gut-brain axis is a major route for neurotropic viruses.

The induction and secretion of IFN, particularly IFN-α and IFN-β, are hallmarks of the innate immune response against viruses (180). Previous studies have shown that infection of dendritic cells and fibroblasts by alphaviruses triggers the activation of the innate immune system via pattern recognition receptors (PRRs) such as Toll-like receptors (TLRs) and RIG-I type receptors (RLRs). This initiates a series of immune responses, including the recruitment of macrophages and other immune cells, and the production of pro-inflammatory cytokines (IL-6 and TNF-α), chemokines (CCL2, CXCL10), and interferons (IFN-α, IFN-β); and signals such as TNF-α and IL-1β, which activate the neuroglial cells, and the activated astrocytes release a variety of molecules such as IL -1β, TNF-α, IL-6, IFN-γ, TGF-β and CCL2, which modulate BBB permeability and promote neuroinflammation (181). BBB dysfunction occurs through cytokine storms increasing membrane permissibility (171). Neuroinflammation is closely associated with BBB destruction and neuroinvasive viral disease.

To the best of our knowledge, some of neuroviruses are able to invade the brain parenchyma via a “Trojan horse” mechanism, through the diapedesis of infected immune cells that either cross the BBB paracellularly or transcellularly (182). The role of immune cells in pathologic neuroinflammation and neurodegeneration in neurotropic viruses infections remains to be further investigated because of the diversity and uncertainty of the origin of immune cells in the CNS.

### 3.2 SARS-CoV-2 infection and neuroinflammation

Theoretically, SARS-CoV-2 infection leads to cellular damage, immune cell activation, and inflammation, which together result in microvascular injury and BBB compromise. By studying the K18-hACE2 infection model, Haowen Qiao et al. reported clear evidence of microvascular damage and breakdown of the BBB, and substantial microglia activation occurred in all three major brain regions after SARS-CoV-2 infection, indicating the coexistence of neuroinflammation and vascular inflammation (183). Preliminary clinical data indicate that SARS-CoV-2 infection is associated with neurological and neuropsychiatric illness; additionally, SARS-CoV-2 infection has been suggested to induce the body to initiate a postinfection-mediated immune response mechanism that ultimately results in autoimmune diseases (184). According to several studies of patients with COVID-19, the CNS shows sustained trends of monocyte expansion and T-cell changes after SARS-CoV-2 infection, and the expansion of monocyte subsets with antiviral and antigen-presenting phenotypes, which are largely mediated by Th1 cytokines, may contribute to BBB disruption and neuroinflammation (185–187).

Laura Pellegrini et al. reported the expression of the viral receptor ACE2 in mature choroid plexus cells expressing abundant lipoproteins, infection with SARS-CoV-2 damages the choroid plexus epithelium, leading to leakage across this important barrier and resulting in pathogens, immune cells and cytokines entering the cerebrospinal fluid and brain (188). Disruption of the barrier may lead to abnormal entry of immune cells and cytokines, which can trigger harmful neuroinflammation. However, previous reports suggest that SARS-CoV-2 is usually not present in the CSF of patients with neurological symptoms arguing against frequent active CNS invasion of the virus (189). Nevertheless, as in other virus infections, considering sample quality, preservation, transportation, handling and technical issues, a negative PCR-test does not exclude the presence of the virus in tissue. Therefore, further studies on antibodies against SARS-CoV-2 would be useful. It’s different from other neurotropic viruses.

Previous evidence indicates that replicative infection of endothelial cells by SARS-CoV-2 has yet to be demonstrated both *in vitro* and *in vivo*, and the limitations of the current public database and available animal models constrain the progress of mechanistic studies revealing how SARS-CoV-2 infection triggers endothelial inflammation and the research on the cellular tropism of SARS-CoV-2 (190). Peng Wang et al. described a microphysiological system integrating alveolus and BBB tissue chips and suggested that systemic inflammation likely contributes to neuropathogenesis following SARS-CoV-2 infection, and that direct viral neural invasion might not be a prerequisite for this neuropathogenesis (191). In this study, compared with direct exposure of chips to SARS-CoV-2, infusion of media from infected chips resulted in more severe damage to the chips, including endothelial dysfunction, pericyte detachment and neuroinflammation (191).

The findings of Juan Prieto-Villalobos et al. indicate that a novel mechanism through which SARS-CoV-2 disrupts cell function, upon SARS-CoV-2 infection, the activation of Cx43 hemichannels by spike S1 occurs rapidly, and this activation leads to an increase in the release of ATP and enhances ATP-mediated [Ca^2+^]_i_ dynamics, and the presence of the SARS-CoV-2 binding receptor ACE2 potentiates this effect (192). These findings provide a new target for identifying the molecular mechanism underlying the neuroinflammation and cytosolic damage caused by SARS-CoV-2 infection.

Cytokines play important roles as mediators of the inflammatory response during viral infections (27). Numerous lines of evidence suggest that a dysregulated immune response, with the release of large amounts of pro-inflammatory cytokines, is involved in the pathogenesis of the severe manifestations of COVID-19 (193). SARS-CoV-2 infection has been confirmed to cause immune cell disorders and cytokine storms in the body (194, 195). These findings suggest that in addition to being directly affected by SARS-CoV-2 infection, indirect mechanisms, such as neuroinflammation secondary to viral infection, may be key in causing neurologic symptoms. In one study, the levels of cytokines such as IL-6, IL-10 and TNF-α significantly increased during SARS-CoV-2 infection but decreased during the recovery period; thus, the expression levels of these cytokines may be directly dependent on active viral replication (196).

Considering the overlapping pathogenesis of different viruses, intraocular inflammation may also occur following SARS-CoV-2 infection (178). Additionally, SARS-CoV-2 can act as a stressor and reactivate other viruses in patients with COVID-19 and prolong COVID-19 symptoms (45).

### 3.3 Pathogenesis associated with neuroinflammation due to SARS-CoV-2 infection

#### 3.3.1 Direct neuronal damage

Previous studies have shown that ACE2 receptors are expressed in glial cells of the brain and spinal cord neurons (197); thus, SARS-CoV-2 can adhere to, replicate in, and directly damage neuronal tissue. Neuroinflammation is a feature of neurodegenerative diseases. Nerve infections are associated with neurodegenerative diseases and neurovascular remodelling, which can cause vascular endothelial dysfunction, leading to circulatory disturbances (198, 199).

A study revealed that patients with severe long COVID-19 exhibited high degrees of peripheral macrophage activation, which disrupted the BBB and damaged tissues (200). The permeability and microvascular injury of the BBB after the addition of extracted SARS-CoV-2 spike protein have been observed in microfluidic models, and it has been speculated that endothelial damage is due to a proinflammatory response (201).

#### 3.3.2 Cytokine storm

In addition to causing critical pulmonary and systemic injuries, SARS-CoV-2 infection is also responsible for cytokine storms (202). There is no doubt that inflammatory cytokines play important roles in the development of viral infection and the amplification of virus invasion.

SARS-CoV-2 infection leads to the production of a variety of immune mediators, such as IL-1β, IL-6, CXCL10, TNFα and other cytokines, which exacerbate the immune response and dysregulate soluble immune mediators, referred to as a “cytokine storm” (195). Increased expression levels of proinflammatory cytokines may exacerbate neuroinflammation. COVID-19 induces the production of inflammatory cytokines that can activate the hypothalamic−pituitary−adrenal axis and indoleamine-2,3-dioxygenase enzymes, which can contribute to neuroglial activation, neuroinflammation, neurotoxicity, and neuronal cell death (203).

After the acute phase, the special immune and inflammatory responses to SARS-CoV-2 infection can continue for months, leading to a state of persistent inflammation with increased levels of chemokines, cytokines and inflammatory molecules. To our knowledge, this prolonged variation in inflammation-related factors has been linked to the activation of some immune cell populations. Owing to chronic stimulation by antigens, T cells express different cytokines, especially those that are more common in CD8^+^ T cells (204). Cytotoxic CD8^+^ T cells can accumulate near the vasculature and generate massive amounts of cytokines that disrupt the BBB, leading to vascular leakage and the propagation of inflammation (205, 206). However, persistent T-cell changes and neurological deficits are related to age (207). Furthermore, some macrophage-derived proteins have been confirmed to stimulate the axonal regeneration of retinal ganglion cells (RGCs) (208), and macrophage inflammatory protein 1β (MIP-1β) has been found to induce both the chemotaxis and adhesion of T cells (209).

Not surprisingly, individual features and the complex interactions between the virus and the patient’s immune system play notable roles in the diversity of clinical manifestations. However, effective immune responses mediated by both T and B lymphocytes are produced in the bodies of patients postinfection, regardless of whether they are asymptomatic or severely infected (210–213). In particular, the numbers of CD4^+^ T and CD8^+^ T lymphocytes are significantly increased, and these cells secrete the antiviral protein interferon-γ (IFN-γ) (213, 214). Similarly, many studies have shown that typical T-cell responses against SARS-CoV-2 increase with increasing viral load, as demonstrated by significant increases in the levels of IFN-γ-producing CD8^+^ T cells in the sera of patients with persistent SARS-CoV-2 PCR positivity (215, 216). One study revealed that increased severity of neurological symptoms was associated with a diminished CD4^+^ T-cell response against the SARS-CoV-2 spike protein (174). The T-cell response is clearly important for diminishing the severity of long-term neurological COVID-19; in addition, COVID-19 mRNA vaccination can increase the ability of T cells to respond (174).

BBB dysfunction is considered the key mechanism underlying long-term COVID-19 complications. With alterations in cytokine activity and glial cell overactivation, ezrin (EZR) levels are increased in patients with long COVID-19, whereas increased nuclear factor-κB (NF-κB) levels cause endothelial cell death and increase extracellular glutamate levels, resulting in BBB disruption and neurodegeneration (200, 217, 218). Moreover, a survey of novel brain organoid models revealed that at 72 hours postinfection, IFN-stimulated gene expression and microglial phagocytosis were upregulated, resulting in engulfment of nerve termini and elimination of synapses (219). The levels of tumour necrosis factor receptor superfamily member 11b (TNFRSF11B), which are also increased in patients with severe long COVID-19, have been confirmed to contribute to neuroinflammatory processes and microglial overstimulation (200). COVID-19 is also known to increase the risk of changes in the CNS, including haemorrhages, ischaemic infarcts and hypoxia, during the acute phase of infection (167, 220). Within this context, there may be intimate associations between ischaemia, hypoxia and neuroinflammation secondary to COVID-19. These changes may develop similarly in ocular tissue at the same time.

#### 3.3.3 Oxidative stress

The level of ROS in organisms is significantly elevated during SARS-CoV-2 infection, and overactivation of oxidative stress and impairment of antioxidant defence mechanisms induce apoptosis (221), with unavoidable effects on viral replication and associated diseases. SARS-CoV-2 increases oxidative stress in neural tissues and promotes neuronal cell death (203, 222).

Thomas Ernst et al. evaluated 29 participants post-COVID-19 who had persistent neuropsychiatric symptom sequelae and reported that lower total N-acetylaspartate (tNAA) and glutamate + glutamine levels indicated neuronal injury, whereas lower myoinositol levels reflected glial cell dysfunction, possibly related to mitochondrial dysfunction and oxidative stress in patients (223).

Owing to its neuroinvasive potential, SARS-CoV-2 infection may increase susceptibility to the development of Parkinson’s disease (PD), as the pathological changes in PD involve oxidative stress, mitochondrial dysfunction, neuroinflammation, and neurodegeneration (224). Oxidative damage to DNA, RNA, proteins, and lipids, along with the depletion of antioxidant enzymes, suggests that oxidative stress plays central roles in the pathophysiology of COVID-19 and PD (225).

#### 3.3.4 Autoantibodies

Autoantibodies play a role in the immune response induced by SARS-CoV-2 infection. Research has revealed that GPCR-AAbs are present in patients with SARS-CoV-2 infection and that GPCR-Aab seropositivity may be related to the loss of retinal capillary microcirculation (128), which may lead to persistent chronic neuroinflammation. Another study showed that neutralization of GPCR-AAbs can increase retinal capillary microcirculation in patients with glaucoma who have recovered from COVID-19 (129).

Anosmia, dysgeusia and headache are common neurological manifestations of acute COVID-19; however, fatigue, cognitive dysfunction (brain fog, memory issues, and attention disorders) and sleep disturbances, the most common neurological symptoms observed in patients with long COVID-19, are caused mostly by prolonged neuroinflammation secondary to innate immune activation (immune cell migration and chemokine release) and humoral activation (autoantibody generation) (226–228). To our knowledge, the autoimmune antibody reaction is suggested to be the product of specific immune and inflammatory reactions rather than being caused by the virus directly (229–233).

**Figure 1:**
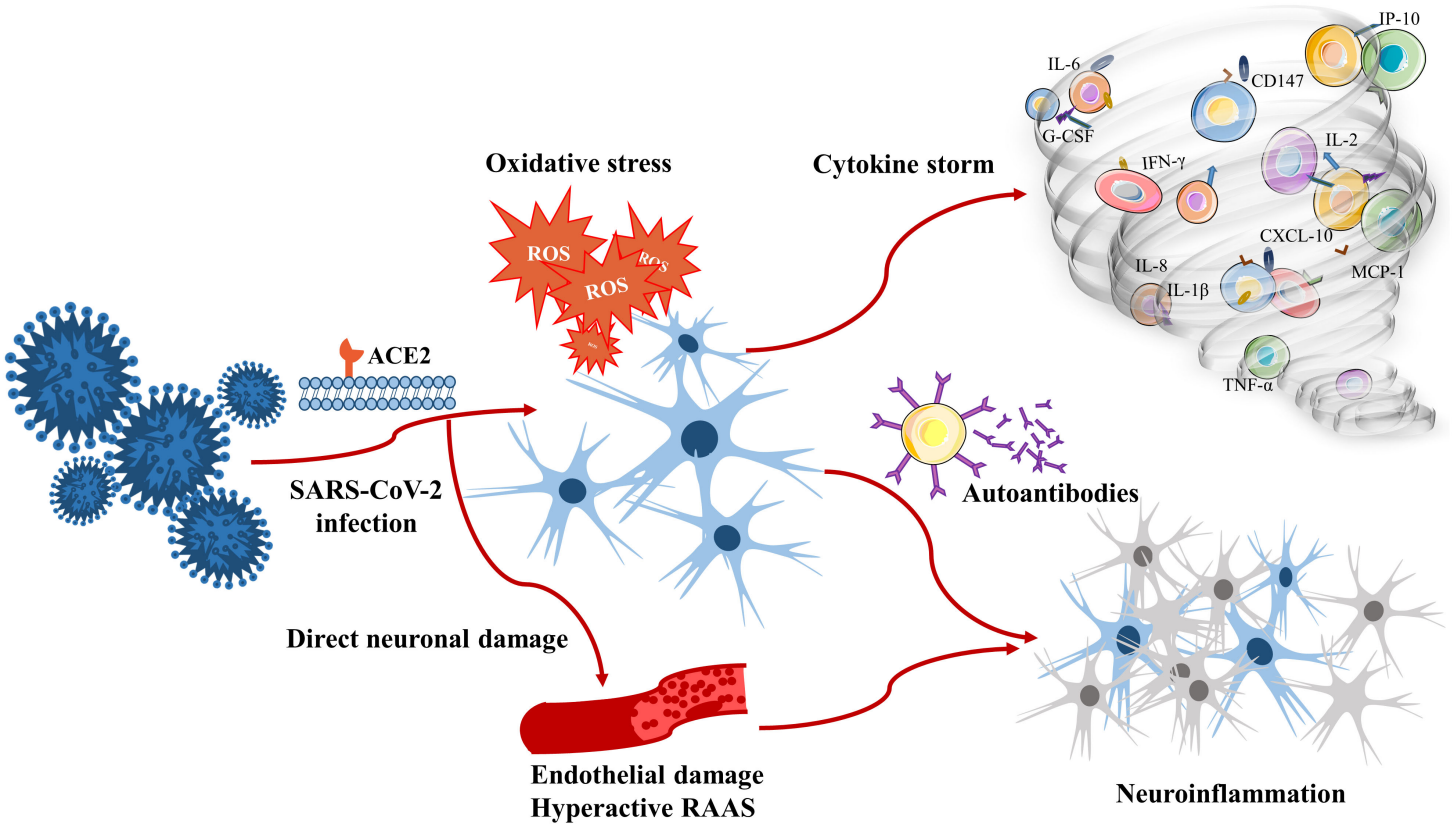
Pathogenesis associated with neuroinflammation due to SARS-CoV-2 infection.

There is evidence that anti-ACE2 antibodies can cause an abnormal renin–angiotensin system response as well as ischaemia associated with malignant hypertension and upregulation of the expression levels of thrombo-inflammatory pathway components (233). It can be presumed that these antibodies are associated with neurological manifestations after SARS-CoV-2 infection, both in the acute postinfection phase and in the chronic persistent infection period. Research supporting autoantibody generation after SARS-CoV-2 infection has focused mostly on case reports and studies, which are limited by sample size, follow-up time constraints and generalizability. Although autoantibodies can drive inflammation, neuronal dysfunction, and neurodegeneration, which are observed in patients with COVID-19, the underlying mechanisms have not been fully identified, and further investigations are needed to elucidate the role of autoantibodies in the pathogenesis of SARS-CoV-2 infection. The possible pathogenesis of neuroinflammation due to SARS-CoV-2 infection is summarized in **Figure 1**.

## 4 Interventions

### 4.1 Antiviral therapy and neuroprotective effects

The main protease (Mprotease) of SARS-CoV-2 is a key target for antiviral drug development. Guilherme Schmitt Rieder et al. reported that the interaction of selenocompounds with Cys145 can contribute to the inhibition of Mprotease and viral replication *in vitro (* 234), which provides new ideas for studying potential inhibitors of viral replication.

According to reports, antiviral drugs, specifically remdesivir, molnupiravir, fluvoxamine and the nirmatrelvir/ritonavir combination (Paxlovid), which are used to treat acute COVID-19, can substantially reduce mortality and hospitalization (235, 236). Cannabidiol has been confirmed to downregulate the key enzymes ACE2 and TMPRSS2, which are involved in SARS-CoV-2 invasion and the potential evolution process (237). Additionally, some of the available literature focuses on neuroprotective effects related to cannabidiol (238, 239), showing that cannabidiol may help alleviate the neurological symptoms of long COVID-19.

Remdesivir shows antiviral activity by binding to and blocking the action of more than one target in SARS-CoV-2, such as the membrane protein (Mprotein), the RNA-dependent RNA polymerase (RDRP), and the Mprotease (240). Bernal et al. suggested that antiviral drugs in combination with glucocorticoids (e.g., remdesivir plus dexamethasone) can reduce the risk of hospitalization and improve the clinical outcome of patients hospitalized with COVID-19 (241).

The authors of another report suggested that disulfiram inhibits viral replication, thereby suppressing acute inflammation and progressive fibrosis; therefore, it is the drug of choice for patients with COVID-19 with elevated early D-dimer concentrations and the development of microangiopathy (242).

### 4.2 Anti-inflammatory therapy and immunotherapy

Many of the findings of available studies that focused on vaccination and medication for patients with COVID-19 broadly suggest that controlling inflammation postinfection can decrease the activation of immune cells and the significant neuroimmune response while reducing the release of subsequent inflammatory cytokines. Therefore, infection-related symptoms can be alleviated (243–247).

It has also been suggested that tetracyclines, including minocycline and doxycycline, have significant neuroprotective and anti-inflammatory properties and may be potential agents for the treatment of SARS-CoV-2-related neuroinflammation (248).

Immunomodulators have extensive anti-inflammatory effects. Among patients suffering from COVID-19 syndrome with dangerous hyperinflammatory conditions, to our knowledge, the findings of many reliable pathophysiological and pharmacological studies have supported the use of therapeutic strategies targeting IL-6 or its receptor (249). For example, the antagonistic blockade of IL-6R by tocilizumab appears to be a viable treatment for patients with severe COVID-19 (249). Furthermore, expectations are especially high for anakinra and baricitinib (214, 250). IL-10, an antifibrotic agent, has been confirmed to exert potent immunomodulatory, anti-inflammatory and antiviral effects (251). Thus, IL-10 is considered another possible treatment agent for COVID-19. In addition, lithium carbonate was shown to reduce the number of days of hospital and intensive care unit admission as well as the risk of death while decreasing inflammatory cytokine levels by preventing cytokine storms and reducing the duration of COVID-19 (252).

Omega-3 polyunsaturated fatty acids (PUFAs) have anti-inflammatory and neuroprotective effects. Ting-Hui Liu et al. reported that omega-3 supplementation significantly reduced the risk of developing psychiatric sequelae post-COVID-19 diagnosis (253), suggesting the potential efficacy of omega-3 PUFAs in alleviating psychiatric sequelae following COVID-19.

César A Zaa et al. reported that natural compounds such as flavonoids, alkaloids, terpenoids, curcumin, and resveratrol could exhibit neuroprotective effects by modulating signalling pathways affected by COVID-19, and among them, curcumin even directly affects the replication cycle of SARS-CoV-2 (254). These findings provide a new basis for the study of neuroprotective compounds to prevent or alleviate neurological damage in patients with COVID-19.

Quercetin treatment successfully reduces the levels of biomarkers indicative of tissue inflammation and infection, such as ferritin, lactate dehydrogenase, D-dimer, and C-reactive protein (CRP) (255). Curcumin nanocapsules can directly reduce the levels of the proinflammatory cytokines IFN-γ and IL-17 and increase the levels of the anti-inflammatory cytokines TGF-β and IL-4 in patients with COVID-19 (256). Vitamin C has antioxidant, antiapoptotic and anti-inflammatory effects; thus, vitamin C supplementation has also been recognized as a potential method for combating COVID-19 (257).

Biomarkers, as measurable biological signals, can reflect the body’s inflammatory response, oxidative stress, immune responses, metabolism and other processes (258). Owing to the complex pathophysiological processes of SARS-CoV-2 postinfectious ocular complications involving multiple molecular pathways, further studies and verifications are needed to select the best representative among the numerous biomarker candidates, which would not only have high specificity and sensitivity but also be convenient to detect.

### 4.3 Future prospects

Adherence to ocular hypotensive medication worsened during the COVID-19 pandemic and seems to be related to patient resilience (259). Notably, there are broad research prospects for improving treatment adherence in patients with ocular diseases in the postpandemic era.

Which anti-inflammatory therapies can both contribute to anti-neuroinflammatory effects in people with SARS-CoV-2 infection and have good CNS penetrance? Future larger basic and clinical trials are needed to provide further evidence that can reveal suitable and reliable biomarkers, predict sensitivity to various antiviral medications and explore a personalized approach for patients with severe COVID-19 manifestations, whether ocular or multisystemic.

## 5 Conclusions

The ocular manifestations of patients with COVID-19 should be taken seriously by ophthalmologists, as these manifestations may be the initial symptoms of these patients. Conjunctivitis, the first manifestation of SARS-CoV-2 infection, has been reported (40). Ocular symptoms are among the common complications after COVID-19. The survival of SARS-CoV-2 in the eyes provides evidence that virus-induced immune disorders and cytokine storms are likely to affect the intraocular environment, thereby triggering an intraocular inflammatory response and causing ocular discomfort in patients while altering intraocular cytokine expression. With the dynamic increase in the SARS-CoV-2 infection rate, the incidence of COVID-19-associated ocular diseases has also increased. Concurrently, the consistent increase in the ageing of the population will continue to increase the prevalence and burden of ocular diseases. The study of COVID-19-related ocular diseases will enable prompt treatment of ocular diseases and provide advanced evidence for COVID-19 pathogenesis investigations, disease duration monitoring and prognosis determination.

Collecting samples from COVID-19 patients is not an easy task and is complicated by safety concerns about collecting samples with infectious pathogens, which poses a considerable challenge for research. Existing explorations are mostly limited to retrospective studies and isolated case reports, with a few prospective explorations, mostly at the cellular level, or the use of animal models. Postmortem examination is also an option. Studies on human *in vivo* samples are lacking. Whether systemic inflammation induced by SARS-CoV-2 itself or by viral infection directly affects the function and survival of cells in different tissues remains a very uncertain aspect, as evidence from large-scale studies is lacking, both in animal models and in human studies. The possibility that the tissue dysfunction and damage observed in COVID-19 patients may depend on the direct action of SARS-CoV-2 viral proteins cannot be excluded. Nevertheless, it is unclear whether the neuroinflammation and subsequent neurotoxicity that develop after SARS-CoV-2 infection are indirectly induced by increased concentrations of cytokines in the local environment and systemic inflammation or are directly driven by viral infection and barrier disruption. Distinguishing between these two potentially interacting mechanisms of action is a daunting challenge that cannot be avoided currently.

However, SARS-CoV-2 neurotropism, vascular endothelial disruption, immunocyte overactivation, inflammatory cytokine generation, and BRB dysfunction may interact with intraocular neuroinflammation secondary to SARS-CoV-2 infection. Do the symptoms and structural features of ocular complications differ in patients with COVID-19? Are there long-term ocular complications in people who recover from SARS-CoV-2 infection? Further basic and clinical studies, larger-scale studies with bigger number of cases and longer follow-up periods are needed to truly understand the natural history of SARS-CoV-2 related neuroinflammation and to determine more uncharted areas of SARS-CoV-2 pathogenesis and progression. Concurrently, well-designed trials of targeted interventions are needed to provide greater clarity on treatment efficacy.

## References

[1] Wu Z McGoogan JM. Characteristics of and important lessons from the coronavirus disease 2019 (COVID-19) outbreak in China: summary of a report of 72 314 cases from the Chinese center for disease control and prevention. JAMA. (2020) 323(13):1239–42. doi: 10.1001/jama.2020.2648 32091533

[2] World Health Organization. data.who.int, WHO Coronavirus (COVID-19) dashboard > More resources [Dashboard]. (2023). Available online at: https://data.who.int/dashboards/covid19/more-resources.

[3] Brann DH Tsukahara T Weinreb C Lipovsek M Van den Berge K Gong B. Non-neuronal expression of SARS-CoV-2 entry genes in the olfactory system suggests mechanisms underlying COVID-19-associated anosmia. Sci Adv. (2020) 6(31):eabc5801. doi: 10.1126/sciadv.abc5801 32937591 PMC10715684

[4] Hoffmann M Kleine-Weber H Schroeder S Krüger N Herrler T Erichsen S. SARS-CoV-2 cell entry depends on ACE2 and TMPRSS2 and is blocked by a clinically proven protease inhibitor. Cell. (2020) 181(2):271–280.e8. doi: 10.1016/j.cell.2020.02.052 32142651 PMC7102627

[5] Katopodis P Kerslake R Davies J Randeva HS Chatha K Hall M. COVID-19 and SARS-CoV-2 host cell entry mediators: Expression profiling of TMRSS4 in health and disease. Int J Mol Med. (2021) 47(4):64. doi: 10.3892/ijmm.2021.4897 33649798 PMC7914073

[6] Bakhshandeh B Sorboni SG Javanmard AR Mottaghi SS Mehrabi MR Sorouri F. Variants in ACE2; potential influences on virus infection and COVID-19 severity. Infect Genet Evol. (2021) 90:104773. doi: 10.1016/j.meegid.2021.104773 33607284 PMC7886638

[7] DiSabato D Quan N Godbout JP. Neuroinflammation: the devil is in the details. J Neurochem. (2016) 139:136–53. doi: 10.1111/jnc.2016.139.issue-S2 PMC5025335 26990767

[8] Hosseini S Wilk E Michaelsen-Preusse K Gerhauser I Baumgärtner W Geffers R. Long-term neuroinflammation induced by influenza A virus infection and the impact on hippocampal neuron morphology and function. J Neurosci. (2018) 38(12):3060–80. doi: 10.1523/JNEUROSCI.1740-17.2018 PMC6596076 29487124

[9] Raison CL Capuron L Miller AH. Cytokines sing the blues: inflammation and the pathogenesis of depression. Trends Immunol. (2006) 27(1):24–31. doi: 10.1016/j.it.2005.11.006 16316783 PMC3392963

[10] Hartlage-Rübsamen M Waniek A Meißner J Morawski M Schilling S Jäger C. Isoglutaminyl cyclase contributes to CCL2-driven neuroinflammation in Alzheimer’s disease. Acta Neuropathol. (2015) 129(4):565–83. doi: 10.1007/s00401-015-1395-2 PMC4366547 25666182

[11] Tansey MG McCoy MK Frank-Cannon TC. Neuroinflammatory mechanisms in Parkinson’s disease: Potential environmental triggers, pathways, and targets for early therapeutic intervention. Exp Neurol. (2007) 208(1):1–25. doi: 10.1016/j.expneurol.2007.07.004 17720159 PMC3707134

[12] Elmakaty I Ferih K Karen O Ouda A Elsabagh A Amarah A. Clinical implications of COVID-19 presence in CSF: systematic review of case reports. Cells. (2022) 11(20):3212. doi: 10.3390/cells11203212 36291083 PMC9600635

[13] Burr T Barton C Doll E Lakhotia A Sweeney M. N-methyl-d-aspartate receptor encephalitis associated with COVID-19 infection in a toddler. Pediatr Neurol. (2021) 114:75–6. doi: 10.1016/j.pediatrneurol.2020.10.002 PMC7546658 33246132

[14] Heald DL Devine IM Smith RL Holsopple SAT Arasmith JL Arnold RW. Opsoclonus after COVID-19 in an infant. Pediatr Neurol. (2021) 117:34. doi: 10.1016/j.pediatrneurol.2020.12.009 33662888 PMC7775259

[15] Allahyari F Hosseinzadeh R Nejad JH Heiat M Ranjbar R. A case report of simultaneous autoimmune and COVID-19 encephalitis. J Neurovirol. (2021) 27(3):504–6. doi: 10.1007/s13365-021-00978-w PMC8075017 33904138

[16] Yoo JE Shin HJ Kang HC Lee JS Kim HD Lee HN. Acute necrotizing myelitis associated with COVID-19. Yonsei Med J. (2023) 64(11):692. doi: 10.3349/ymj.2023.0202 37880851 PMC10613760

[17] Stoian A Bajko Z Stoian M Cioflinc RA Niculescu R Arbana?i EM. The occurrence of acute disseminated encephalomyelitis in SARS-coV-2 infection/vaccination: our experience and a systematic review of the literature. Vaccines (Basel). (2023) 11(7):1225. doi: 10.3390/vaccines11071225 37515041 PMC10385010

[18] Baştan B Erdağ Turgeon E Şanlı E Bayar MD Şişman AB Atacan Yaşgüçlükal M. Increased neuropil antibody prevalence in COVID-19 patients with acute ischemic stroke. Neurol Res. (2023) 45(11):988–93. doi: 10.1080/01616412.2023.2252282 37634189

[19] Rosca EC Bilavu R Cornea A Simu M. Chorea following SARS-CoV-2 infection and vaccination: a systematic review of reported cases. Int J Infect Dis. (2023) 134:256–60. doi: 10.1016/j.ijid.2023.07.001 37423421

[20] Brola W Wilski M. Neurological consequences of COVID-19. Pharmacol Rep. (2022) 74(6):1208–22. doi: 10.1007/s43440-022-00424-6 PMC9524739 36180640

[21] Nersesjan V Amiri M Lebech AM Roed C Mens H Russell L. Central and peripheral nervous system complications of COVID-19: a prospective tertiary center cohort with 3-month follow-up. J Neurol. (2021) 268(9):3086–104. doi: 10.1007/s00415-020-10380-x PMC7803470 33438076

[22] Trost A Motloch K Bruckner D Schroedl F Bogner B Kaser-Eichberger A. Time-dependent retinal ganglion cell loss, microglial activation and blood-retina-barrier tightness in an acute model of ocular hypertension. Exp Eye Res. (2015) 136:59–71. doi: 10.1016/j.exer.2015.05.010 26001526

[23] Naskar R Wissing M Thanos S. Detection of early neuron degeneration and accompanying microglial responses in the retina of a rat model of glaucoma. Invest Ophthalmol Vis Sci. (2002) 43(9):2962–8. 12202516

[24] Chidlow G Ebneter A Wood JPM Casson RJ. Evidence supporting an association between expression of major histocompatibility complex II by microglia and optic nerve degeneration during experimental glaucoma. J Glaucoma. (2016) 25(8):681–91. doi: 10.1097/IJG.0000000000000447 27253969

[25] Brochard V Combadière B Prigent A Laouar Y Perrin A Beray-Berthat V. Infiltration of CD4+ lymphocytes into the brain contributes to neurodegeneration in a mouse model of Parkinson disease. J Clin Invest. (2009) 119(1):182–92. doi: 10.1172/JCI36470 PMC2613467 19104149

[26] Ng HW Scott DAR Danesh-Meyer HV Smith JR McGhee CN Niederer RL. Ocular manifestations of COVID-19. Prog Retinal Eye Res. (2024) 102:101285. doi: 10.1016/j.preteyeres.2024.101285 38925508

[27] Niedźwiedź A Kawa M Pius-Sadowska E Kuligowska A Ziontkowska A Wrzałek D. Increased proinflammatory cytokines in tears correspond with conjunctival SARS-CoV-2 positivity in symptomatic COVID-19 patients. Sci Rep. (2022) 12(1):7225. doi: 10.1038/s41598-022-11285-7 35508669 PMC9068775

[28] Zhou X Zhou YN Ali A Liang C Ye Z Chen X. Case report: A re-positive case of SARS-coV-2 associated with glaucoma. Front Immunol. (2021) 12:701295. doi: 10.3389/fimmu.2021.701295 34394095 PMC8355891

[29] Seah I Agrawal R. Can the coronavirus disease 2019 (COVID-19) affect the eyes? A review of coronaviruses and ocular implications in humans and animals. Ocul Immunol Inflammation. (2020) 28(3):391–5. doi: 10.1080/09273948.2020.1738501 PMC7103678 32175797

[30] Li JPO Lam DSC Chen Y Ting DSW. Novel Coronavirus disease 2019 (COVID-19): The importance of recognising possible early ocular manifestation and using protective eyewear. Br J Ophthalmol. (2020) 104(3):297–8. doi: 10.1136/bjophthalmol-2020-315994 32086236

[31] Salducci M La Torre G. COVID-19 emergency in the cruise’s ship: a case report of conjunctivitis. Clin Ter. (2020) 171(3):e189–91. doi: 10.7417/CT.2020.2212 32323704

[32] Chen L Liu M Zhang Z Qiao K Huang T Chen M. Ocular manifestations of a hospitalised patient with confirmed 2019 novel coronavirus disease. Br J Ophthalmol. (2020) 104(6):748–51. doi: 10.1136/bjophthalmol-2020-316304 PMC7211077 32265202

[33] Invernizzi A Pellegrini M Messenio D Cereda M Olivieri P Brambilla AM. Impending central retinal vein occlusion in a patient with coronavirus disease 2019 (COVID-19). Ocular Immunol Inflammation. (2020) 28(8):1290–2. doi: 10.1080/09273948.2020.1807023 32976055

[34] Wu P Duan F Luo C Liu Q Qu X Liang L. Characteristics of ocular findings of patients with coronavirus disease 2019 (COVID-19) in Hubei province, China. JAMA Ophthalmol. (2020) 138(5):575–8. doi: 10.1001/jamaophthalmol.2020.1291 PMC7110919 32232433

[35] Inomata T Kitazawa K Kuno T Sung J Nakamura M Iwagami M. Clinical and prodromal ocular symptoms in coronavirus disease: A systematic review and meta-analysis. Invest Ophthalmol Vis Sci. (2020) 61(10):29. doi: 10.1167/iovs.61.10.29 PMC7441339 32797198

[36] Tostmann A Bradley J Bousema T Yiek WK Holwerda M Bleeker-Rovers C. Strong associations and moderate predictive value of early symptoms for SARS-CoV-2 test positivity among healthcare workers, the Netherlands, March 2020. Eurosurveillance. (2020) 25(16):2000508. doi: 10.2807/1560-7917.ES.2020.25.16.2000508 32347200 PMC7189649

[37] Hong N Yu W Xia J Shen Y Yap M Han W. Evaluation of ocular symptoms and tropism of SARS-CoV-2 in patients confirmed with COVID-19. Acta Ophthalmol. (2020) 98(5):e649–55. doi: 10.1111/aos.14445 PMC7267628 32336042

[38] Yuhang Z Xiaohang X Fengyan Z. Current status of posterior segment lesions associated with novel coronavirus infection. Int Ophthalmol Rev. (2022) 46(1):167–72. doi: 10.3760/cma.j.issn.0253-2727.2019.01.010

[39] Public Health Ophthalmology Branch of Chinese Preventive Medicine Association. Chinese expert consensus on prevention and control of COVID-19 eye disease (2022). Zhonghua Yan Ke Za Zhi. (2022) 58(3):176–81. doi: 10.3760/cma.j.cn112142-20211124-00561 35280024

[40] Ya Y Yanping S Ming Y Cheng H Xiao C Juan Y. Novel coronavirus pneumonia combined with conjunctivitis: three cases report. Chin J Exp Ophthalmol. (2020) 38(11):242–4. doi: 10.3760/cma.j.issn.2095-0160.2020.0006

[41] Yan Y Diao B Liu Y Zhang W Wang G Chen X. Severe acute respiratory syndrome coronavirus 2 nucleocapsid protein in the ocular tissues of a patient previously infected with coronavirus disease 2019. JAMA Ophthalmol. (2020) 138(11):1201–4. doi: 10.1001/jamaophthalmol.2020.3962 33034620 PMC7545349

[42] Parvez Y AlZarooni F Khan F. Optic neuritis in a child with COVID-19: A rare association. Cureus. 13(3):e14094. doi: 10.7759/cureus.14094 PMC8075757 33927916

[43] Hsia NY Hsu AY Wang YH Li JX Chen HS Wei JC. The risk assessment of uveitis after COVID-19 diagnosis: A multicenter population-based study. J Med Virol. (2023) 95(10):e29188. doi: 10.1002/jmv.v95.10 37881132

[44] Romero-Castro RM Ruiz-Cruz M Alvarado-De La Barrera C González-Cannata MG Luna-Villalobos YA García-Morales AK. Posterior segment ocular findings in critically ill patients with COVID-19. Retina. (2022) 42(4):628–33. doi: 10.1097/IAE.0000000000003457 35350045

[45] Leng A Shah M Ahmad SA Premraj L Wildi K Li Bassi G. Pathogenesis underlying neurological manifestations of long COVID syndrome and potential therapeutics. Cells. (2023) 12(5):816. doi: 10.3390/cells12050816 36899952 PMC10001044

[46] Mina Y Enose-Akahata Y Hammoud DA Videckis AJ Narpala SR O'Connell SE. Deep phenotyping of neurologic postacute sequelae of SARS-coV-2 infection. Neurol Neuroimmunol Neuroinflamm. (2023) 10(4):e200097. doi: 10.1212/NXI.0000000000200097 37147136 PMC10162706

[47] Han L Huizhen C. Ocular manifestations of novel coronavirus pneumonia. Int J Ophthalmol. (2022) 22(12):2105–10. doi: 10.3980/j.issn.1672-5123.2022.12.34

[48] Dinkin M Gao V Kahan J Bobker S Simonetto M Wechsler P. COVID-19 presenting with ophthalmoparesis from cranial nerve palsy. Neurology. (2020) 95(5):221–3. doi: 10.1212/WNL.0000000000009700 32358218

[49] Restivo DA Centonze D Alesina A Marchese-Ragona R. Myasthenia gravis associated with SARS-coV-2 infection. Ann Internal Med. (2020) 173(12):1027–8. doi: 10.7326/L20-0845 PMC7429993 32776781

[50] Huber M Rogozinski S Puppe W Framme C Höglinger G Hufendiek K. Postinfectious onset of myasthenia gravis in a COVID-19 patient. Front Neurol. (2020) 11:576153. doi: 10.3389/fneur.2020.576153 33123081 PMC7573137

[51] de Oliveira RdeMC Santos DH Olivetti BC Takahashi JT. Bilateral trochlear nerve palsy due to cerebral vasculitis related to COVID-19 infection. Arq Neuro-Psiquiatr. (2020) 78(6):385–6. doi: 10.1590/0004-282x20200052 32609196

[52] Monu M Ahmad F Olson RM Balendiran V Singh PK. SARS-CoV-2 infects cells lining the blood-retinal barrier and induces a hyperinflammatory immune response in the retina via systemic exposure. PloS Pathog. (2024) 20(4):e1012156. doi: 10.1371/journal.ppat.1012156 38598560 PMC11034659

[53] Gao J Guo Z Li W Zhang X Zhang XE Cui Z. Ebola virus disrupts the inner blood-retinal barrier by induction of vascular endothelial growth factor in pericytes. PloS Pathog. (2023) 19(1):e1011077. doi: 10.1371/journal.ppat.1011077 36652443 PMC9847965

[54] Xu X Chen P Wang J Feng J Zhou H Li X. Evolution of the novel coronavirus from the ongoing Wuhan outbreak and modeling of its spike protein for risk of human transmission. Sci China Life Sci. (2020) 63(3):457–60. doi: 10.1007/s11427-020-1637-5 PMC7089049 32009228

[55] Zhou P Yang XL Wang XG Hu B Zhang L Zhang W. A pneumonia outbreak associated with a new coronavirus of probable bat origin. Nature. (2020) 579(7798):270–3. doi: 10.1038/s41586-020-2012-7 PMC7095418 32015507

[56] Walls AC Park YJ Tortorici MA Wall A McGuire AT Veesler D. Structure, function, and antigenicity of the SARS-coV-2 spike glycoprotein. Cell. (2020) 181(2):281–292.e6. doi: 10.1016/j.cell.2020.02.058 32155444 PMC7102599

[57] Chen X Yu H Mei T Chen B Chen L Li S. SARS-CoV-2 on the ocular surface: is it truly a novel transmission route? Br J Ophthalmol. (2021) 105(9):1190–5. doi: 10.1136/bjophthalmol-2020-316263 PMC8380887 32788324

[58] Senanayake Pd Drazba J Shadrach K Milsted A Rungger-Brandle E Nishiyama K. Angiotensin II and its receptor subtypes in the human retina. Invest Ophthalmol Vis Sci. (2007) 48(7):3301. doi: 10.1167/iovs.06-1024 17591902

[59] Wagner J Jan Danser AH Derkx FH de Jong TV Paul M Mullins JJ. Demonstration of renin mRNA, angiotensinogen mRNA, and angiotensin converting enzyme mRNA expression in the human eye: evidence for an intraocular renin-angiotensin system. Br J Ophthalmol. (1996) 80(2):159–63. doi: 10.1136/bjo.80.2.159 PMC505409 8814748

[60] Lujia Z Xiuxiu J Bo L. Distribution and clinical significance of ACE2, a key receptor of 2019-nCoV, in ocular tissues. Chin J Exp Ophthalmol. (2020) 38(5):463–7. doi: 10.3760/cma.j.cn115989-20200316-00173

[61] Jeong GU Kwon HJ Ng WH Liu X Moon HW Yoon GY. Ocular tropism of SARS-CoV-2 in animal models with retinal inflammation via neuronal invasion following intranasal inoculation. Nat Commun. (2022) 13(1):7675. doi: 10.1038/s41467-022-35225-1 36509737 PMC9743116

[62] Sen HN Vannella KM Wang Y Chung JY Kodati S Ramelli SC. Histopathology and SARS-coV-2 cellular localization in eye tissues of COVID-19 autopsies. Am J Pathol. (2023) 193(11):1809–16. doi: 10.1016/j.ajpath.2023.02.016 PMC10032059 36963628

[63] Ruiz-Cruz M Ávila-Rios S Ormsby CE Ablanedo-Terrazas Y Alvarado-de la Barrera C Kuri-Cervantes L. Cytokine profiles in aqueous humor and plasma of HIV-infected individuals with ocular syphilis or cytomegalovirus retinitis. Ocular Immunol Inflammation. (2018) 26(1):74–81. doi: 10.1080/09273948.2016.1268170 28081374

[64] Sanghi P Malik M Hossain IT Manzouri B. Ocular complications in the prone position in the critical care setting: the COVID-19 pandemic. J Intensive Care Med. (2021) 36(3):361–72. doi: 10.1177/0885066620959031 32985317

[65] Scalinci SZ Trovato Battagliola E. Conjunctivitis can be the only presenting sign and symptom of COVID-19. IDCases. (2020) 20:e00774. doi: 10.1016/j.idcr.2020.e00774 32373467 PMC7195291

[66] Alnajjar M Al-Mashdali A Nefatti N. COVID-19 case presented initially with keratoconjunctivitis: A case report. Ann Med Surg (Lond). (2021) 71:102957. doi: 10.1016/j.amsu.2021.102957 34691451 PMC8520444

[67] Otaif W Al Somali AI Al Habash A. Episcleritis as a possible presenting sign of the novel coronavirus disease: A case report. Am J Ophthalmol Case Rep. (2020) 20:100917. doi: 10.1016/j.ajoc.2020.100917 32923742 PMC7476899

[68] Nasiri N Sharifi H Bazrafshan A Noori A Karamouzian M Sharifi A. Ocular manifestations of COVID-19: A systematic review and meta-analysis. J Ophthal Vis Res. (2021) 16(1):103–12. doi: 10.18502/jovr.v16i1.8256 PMC7841281 33520133

[69] Ma N Li P Wang X Yu Y Tan X Chen P. Ocular manifestations and clinical characteristics of children with laboratory-confirmed COVID-19 in Wuhan, China. JAMA Ophthalmol. (2020) 138(10):1079–86. doi: 10.1001/jamaophthalmol.2020.3690 PMC7450405 32845280

[70] Zhong Y Wang K Zhu Y Lyu D Yu Y Li S. Ocular manifestations in COVID-19 patients: A systematic review and meta-analysis. Travel Med Infect Dis. (2021) 44:102191. doi: 10.1016/j.tmaid.2021.102191 34763068 PMC8574127

[71] Zhou Y Liu J Cui Y Zhu H Lu Z. Moisture chamber versus lubrication for corneal protection in critically ill patients: A meta-analysis. Cornea. (2014) 33(11):1179. doi: 10.1097/ICO.0000000000000224 25170579

[72] Bhatraju PK Ghassemieh BJ Nichols M Kim R Jerome KR Nalla AK. Covid-19 in critically ill patients in the Seattle region — Case series. N Engl J Med. (2020) 382(21):2012–22. doi: 10.1056/NEJMoa2004500 PMC7143164 32227758

[73] Cook DJ Marshall JC Fowler RA. Critical illness in patients with COVID-19: mounting an effective clinical and research response. JAMA. (2020) 323(16):1559–60. doi: 10.1001/jama.2020.5775 32250408

[74] Xuejie L Ming W Changzheng C Anhuai Y Wei J. Ophthalmologists’ strategy for the prevention and control of coronavirus pneumonia with conjunctivitis or with conjunctivitis as the first symptom. Chin J Exp Ophthalmol. (2020) 38(03):276–80. doi: 10.3760/cma.j.issn.2095-0160.2020.0002

[75] Lu R Zhao X Li J Niu P Yang B Wu H. Genomic characterisation and epidemiology of 2019 novel coronavirus: implications for virus origins and receptor binding. Lancet. (2020) 395(10224):565–74. doi: 10.1016/S0140-6736(20)30251-8 PMC7159086 32007145

[76] Fakih D Zhao Z Nicolle P Reboussin E Joubert F Luzu J. Chronic dry eye induced corneal hypersensitivity, neuroinflammatory responses, and synaptic plasticity in the mouse trigeminal brainstem. J Neuroinflamm. (2019) 16(1):268. doi: 10.1186/s12974-019-1656-4 PMC6918709 31847868

[77] Launay PS Reboussin E Liang H Kessal K Godefroy D Rostene W. Ocular inflammation induces trigeminal pain, peripheral and central neuroinflammatory mechanisms. Neurobiol Dis. (2016) 88:16–28. doi: 10.1016/j.nbd.2015.12.017 26747211

[78] Sudre CH Murray B Varsavsky T Graham MS Penfold RS Bowyer RC. Attributes and predictors of long COVID. Nat Med. (2021) 27(4):626–31. doi: 10.1038/s41591-021-01292-y PMC7611399 33692530

[79] Bai F Tomasoni D Falcinella C Barbanotti D Castoldi R Mulè G. Female gender is associated with long COVID syndrome: a prospective cohort study. Clin Microbiol Infect. (2022) 28(4):611.e9–611.e16. doi: 10.1016/j.cmi.2021.11.002 PMC8575536 34763058

[80] Taquet M Dercon Q Luciano S Geddes JR Husain M Harrison PJ. Incidence, co-occurrence, and evolution of long-COVID features: A 6-month retrospective cohort study of 273,618 survivors of COVID-19. PloS Med. (2021) 18(9):e1003773. doi: 10.1371/journal.pmed.1003773 34582441 PMC8478214

[81] Parotto M Myatra SN Munblit D Elhazmi A Ranzani OT Herridge MS. Recovery after prolonged ICU treatment in patients with COVID-19. Lancet Respir Med. (2021) 9(8):812–4. doi: 10.1016/S2213-2600(21)00318-0 PMC8280055 34273267

[82] Yoo SM Liu TC Motwani Y Sim MS Viswanathan N Samras N. Factors associated with post-acute sequelae of SARS-coV-2 (PASC) after diagnosis of symptomatic COVID-19 in the inpatient and outpatient setting in a diverse cohort. J Gen Intern Med. (2022) 37(8):1988–95. doi: 10.1007/s11606-022-07523-3 PMC8989256 35391623

[83] Iosifescu AL Hoogenboom WS Buczek AJ Fleysher R Duong TQ. New-onset and persistent neurological and psychiatric sequelae of COVID-19 compared to influenza: A retrospective cohort study in a large New York City healthcare network. Int J Methods Psychiatr Res. (2022) 31(3):e1914. doi: 10.1002/mpr.v31.3 35706352 PMC9349863

[84] Gayton JL. Etiology, prevalence, and treatment of dry eye disease. Clin Ophthalmol. (2009) 3:405–12. doi: 10.2147/OPTH.S5555 PMC2720680 19688028

[85] Vergroesen JE Kaynak A Aribas E Kavousi M van Meurs JBJ Klaver CCW. Higher testosterone is associated with open-angle glaucoma in women: a genetic predisposition? Biol Sex Dif. (2023) 14(1):27. doi: 10.1186/s13293-023-00512-z PMC10170716 37161452

[86] Wang Y Cun Q Li J Shen W Yang WY Tao YJ. Prevalence, ethnic differences and risk factors of primary angle-closure glaucoma in a multiethnic Chinese adult population: the Yunnan Minority Eye Study. Br J Ophthalmol. (2023) 107(5):677–82. doi: 10.1136/bjophthalmol-2021-320241 34933895

[87] Gaur S Sindhu N Singh DV Bhattacharya M Sharma A Shinde D. COVID-19-related bilateral acute de-pigmentation of iris with ocular hypertension. Indian J Ophthalmol. (2022) 70(8):3136–9. doi: 10.4103/ijo.IJO_75_22 PMC9672727 35918989

[88] Soydan A Kaymaz A. Bilateral acute depigmentation of the iris determined in two cases after COVID-19 infection. Indian J Ophthalmol. (2023) 71(3):1030. doi: 10.4103/ijo.IJO_949_22 36872735 PMC10229917

[89] Lončarić K Tadić R Radmilović M Vatavuk Z. Bilateral acute iris transillumination (BAIT): A rare syndrome possibly associated with COVID-19 and moxifloxacin use. A Rep 2 Cases. Semin Ophthalmol. (2023) 38(3):312–5. doi: 10.1080/08820538.2023.2168491 36653737

[90] Yagci BA Atas F Kaya M Arikan G. COVID-19 associated bilateral acute iris transillumination. Ocular Immunol Inflammation. (2021) 29(4):719–21. doi: 10.1080/09273948.2021.1933073 34124990

[91] Kuo IC Mostafa HH. Detection of SARS-CoV-2 RNA in the corneal epithelium of a patient after recovery from COVID-19. Am J Ophthalmol Case Rep. (2021) 22:101074. doi: 10.1016/j.ajoc.2021.101074 33748538 PMC7962916

[92] Krawitz BD Sirinek P Doobin D Nanda T Ghiassi M Horowitz JD. The challenge of managing bilateral acute angle-closure glaucoma in the presence of active SARS-coV-2 infection. J Glaucoma. (2021) 30(3):e50. doi: 10.1097/IJG.0000000000001763 33337718

[93] Jasti M Nalleballe K Dandu V Onteddu S. A review of pathophysiology and neuropsychiatric manifestations of COVID-19. J Neurol. (2021) 268(6):2007–12. doi: 10.1007/s00415-020-09950-w PMC7268182 32494854

[94] Kubota T Kuroda N Sone D. Neuropsychiatric aspects of long COVID: A comprehensive review. Psychiatry Clin Neurosci. (2023) 77(2):84–93. doi: 10.1111/pcn.13508 36385449 PMC10108156

[95] Özmen S Özkan Aksoy N Çakır B Alagöz G. Acute angle-closure glaucoma concurrent with COVID 19 infection; case report. Eur J Ophthalmol. (2023) 33(4):NP42–5. doi: 10.1177/11206721221113201 35815850

[96] Nerlikar RR Palsule AC Vadke S. Bilateral acute angle closure glaucoma after prone position ventilation for COVID-19 pneumonia. J Glaucoma. (2021) 30(8):e364–6. doi: 10.1097/IJG.0000000000001864 PMC8366511 33927149

[97] Barosco G Morbio R Chemello F Tosi R Marchini G. Bilateral angle-closure during hospitalization for coronavirus disease-19 (COVID-19): A case report. Eur J Ophthalmol. (2022) 32(3):NP75–82. doi: 10.1177/11206721211012197 PMC9111899 33885335

[98] Lee WS Parsons S Cugley D Rogers S Lim LL Hall A. Increased incidence of glaucoma medication usage in middle-aged Australian males taking antiretroviral medication – a population-based study. J Ophthal Inflammation Infect. (2020) 10(1):30. doi: 10.1186/s12348-020-00218-y PMC7609505 33141357

[99] Lee JW Lee JE Choi HY Lee JS. Oseltamivir (Tamiflu)-induced bilateral acute angle closure glaucoma and transient myopia. Indian J Ophthalmol. (2014) 62:1165–7. doi: 10.4103/0301-4738.109531 PMC4313501 23571265

[100] Yazdani S Esfandiari H Safi S Fatemi A. Oseltamivir (Tamiflu)-induced bilateral ciliochoroidal effusion and angle closure glaucoma: what type of idiosyncratic reaction? J Ophthal Vis Res. (2017) 12(4):434–6. doi: 10.4103/jovr.jovr_200_15 PMC5644413 29090056

[101] Wang L Wei X. T cell-mediated autoimmunity in glaucoma neurodegeneration. Front Immunol. (2021) 12:803485. doi: 10.3389/fimmu.2021.803485 34975917 PMC8716691

[102] Gramlich OW Beck S von Thun Und Hohenstein-Blaul N Boehm N Ziegler A Vetter JM. Enhanced insight into the autoimmune component of glaucoma: IgG autoantibody accumulation and pro-inflammatory conditions in human glaucomatous retina. PloS One. (2013) 8(2):e57557. doi: 10.1371/journal.pone.0057557 23451242 PMC3581473

[103] Holappa M Valjakka J Vaajanen A. Angiotensin(1-7) and ACE2, “The hot spots” of renin-angiotensin system, detected in the human aqueous humor. Open Ophthalmol J. (2015) 9:28–32. doi: 10.2174/1874364101509010028 25926900 PMC4407001

[104] Foureaux G Nogueira JC Nogueira BS Fulgêncio GO Menezes GB Fernandes SO. Antiglaucomatous effects of the activation of intrinsic angiotensin-converting enzyme 2. Invest Ophthalmol Vis Sci. (2013) 54(6):4296–306. doi: 10.1167/iovs.12-11427 PMC3739491 23702784

[105] Gheblawi M Wang K Viveiros A Nguyen Q Zhong JC Turner AJ. Angiotensin-converting enzyme 2: SARS-coV-2 receptor and regulator of the renin-angiotensin system. Circ Res. (2020) 126(10):1456–74. doi: 10.1161/CIRCRESAHA.120.317015 PMC7188049 32264791

[106] Muslim S Nasrin N Alotaibi FO Prasad G Singh SK Alam I. Treatment options available for COVID-19 and an analysis on possible role of combination of rhACE2, angiotensin (1-7) and angiotensin (1-9) as effective therapeutic measure. SN Compr Clin Med. (2020) 2(10):1761–6. doi: 10.1007/s42399-020-00407-9 PMC7442548 32864572

[107] Miura Y Ohkubo H Nakano A Bourke JE Kanazawa S. Pathophysiological conditions induced by SARS-CoV-2 infection reduce ACE2 expression in the lung. Front Immunol. (2022) 13:1028613. doi: 10.3389/fimmu.2022.1028613 36405683 PMC9673245

[108] Chen Y Yan H Li G Zhang Y. Higher TGF-β1, TGF-β2, MMP-2, and TIMP-1 levels in the aqueous humor of patients with acute primary angle closure. Ophthal Res. (2021) 64(1):62–7. doi: 10.1159/000507762 32259818

[109] Bell K von Thun N Teister J Grus F. Modulation of the immune system for the treatment of glaucoma. Curr Neuropharmacol. (2018) 16(7):942–58. doi: 10.2174/1570159X15666170720094529 PMC6120111 28730968

[110] Mélik Parsadaniantz S Réaux-le Goazigo A Sapienza A Habas C Baudouin C. Glaucoma: A degenerative optic neuropathy related to neuroinflammation? Cells. (2020) 9(3):535. doi: 10.3390/cells9030535 32106630 PMC7140467

[111] Wei X Cho KS Thee EF Jager MJ Chen DF. Neuroinflammation and microglia in glaucoma: time for a paradigm shift. J Neurosci Res. (2019) 97(1):70–6. doi: 10.1002/jnr.24256 PMC6239948 29775216

[112] Weinreb RN Aung T Medeiros FA. The pathophysiology and treatment of glaucoma. JAMA. (2014) 311(18):1901–11. doi: 10.1001/jama.2014.3192 PMC4523637 24825645

[113] Ahmad F Deshmukh N Webel A Johnson S Suleiman A Mohan RR. Viral infections and pathogenesis of glaucoma: a comprehensive review. Clin Microbiol Rev. (2023) 0(4):e00057–23. doi: 10.1128/cmr.00057-23 PMC10870729 37966199

[114] Sng CCA Ang M Barton K. Chapter 13 - Uveitis and glaucoma: new insights in the pathogenesis and treatment. Prog Brain Res. (2015) 221:243–69. doi: 10.1016/bs.pbr.2015.06.008 26518082

[115] Soman M Indurkar A George T Sheth JU Nair U. Rapid onset neovascular glaucoma due to COVID-19-related retinopathy. J Curr Glaucoma Pract. (2022) 16(2):136–40. doi: 10.5005/jp-journals-10078-1356 PMC9452705 36128075

[116] de-la-Torre A Valdes-Camacho J Foster CS. Bilateral herpes simplex uveitis: review of the literature and own reports. Ocular Immunol Inflammation. (2017) 25(4):497–502. doi: 10.3109/09273948.2016.1142572 27003735

[117] Neumann R Barequet D Rosenblatt A Amer R Ben-Arie-Weintrob Y Hareuveni-Blum T. Herpetic anterior uveitis – analysis of presumed and PCR proven cases. Ocular Immunol Inflammation. (2019) 27(2):211–8. doi: 10.1080/09273948.2018.1483521 30311824

[118] Ying Y Zhai R Sun Y Sheng Q Fan X Kong X. The occurrence of acute primary angle closure triggered, aggravated, and accelerated by COVID-19 infection: retrospective observational study. Front Public Health. (2023) 11:1196202. doi: 10.3389/fpubh.2023.1196202 37645709 PMC10461000

[119] Elnahry AG Asal ZB Shaikh N Dennett K Abd Elmohsen MN Elnahry GA. Optic neuropathy after COVID-19 vaccination: a report of two cases. Int J Neurosci. (2023) 133(8):901–7. doi: 10.1080/00207454.2021.2015348 34906029

[120] Cavalcanti DD Raz E Shapiro M Dehkharghani S Yaghi S Lillemoe K. Cerebral venous thrombosis associated with COVID-19. AJNR Am J Neuroradiol. (2020) 41(8):1370–6. doi: 10.3174/ajnr.A6644 PMC7658892 32554424

[121] Bosello F Marastoni D Pizzini FB Zaffalon C Zuliani A Turri G. Atypical myelin oligodendrocyte glycoprotein antibody–associated optic neuritis and acute demyelinating polyneuropathy after SARS-CoV-2 infection: Case report and literature review. J Neuroimmunol. (2023) 375:578011. doi: 10.1016/j.jneuroim.2022.578011 36621074 PMC9779985

[122] Sheth JU Narayanan R Goyal J Goyal V. Retinal vein occlusion in COVID-19: A novel entity. Indian J Ophthalmol. (2020) 68(10):2291. doi: 10.4103/ijo.IJO_2380_20 32971697 PMC7727974

[123] Walinjkar JA Makhija SC Sharma HR Morekar SR Natarajan S. Central retinal vein occlusion with COVID-19 infection as the presumptive etiology. Indian J Ophthalmol. (2020) 68(11):2572. doi: 10.4103/ijo.IJO_2575_20 33120696 PMC7774137

[124] Nguyen NCI Eandi C Guex-Crosier Y. Central retinal vein occlusion after COVID-19 infection. Klin Monbl Augenheilkd. (2023) 240(4):509–13. doi: 10.1055/a-2040-3653 37164394

[125] Shahri SHG Abrishami M Shayanfar H Khazaei S. Bilateral anterior ischemic optic neuropathy and choroidal ischemia in a patient with COVID-19 infection. Clin Case Rep. (2023) 11(1):e6834. doi: 10.1002/ccr3.v11.1 36703768 PMC9869642

[126] Teo KY Invernizzi A Staurenghi G Cheung CMG. COVID-19-related retinal micro-vasculopathy – A review of current evidence. Am J Ophthalmol. (2022) 235:98–110. doi: 10.1016/j.ajo.2021.09.019 34587494 PMC8465265

[127] Jidigam VK Singh R Batoki JC Milliner C Sawant OB Bonilha VL. Histopathological assessments reveal retinal vascular changes, inflammation, and gliosis in patients with lethal COVID-19. Graefes Arch Clin Exp Ophthalmol. (2022) 260(4):1275–88. doi: 10.1007/s00417-021-05460-1 PMC8553591 34714382

[128] Szewczykowski C Mardin C Lucio M Wallukat G Hoffmanns J Schröder T. Long COVID: association of functional autoantibodies against G-protein-coupled receptors with an impaired retinal microcirculation. Int J Mol Sci. (2022) 23(13):7209. doi: 10.3390/ijms23137209 35806214 PMC9266742

[129] Hohberger B Harrer T Mardin C Kruse F Hoffmanns J Rogge L. Case report: neutralization of autoantibodies targeting G-protein-coupled receptors improves capillary impairment and fatigue symptoms after COVID-19 infection. Front Med. (2021) 8. doi: 10.3389/fmed.2021.754667 PMC8637609 34869451

[130] Perilli L Fetta M Capponi M Guido CA Grosso S Iannetti P. Peripheral nervous system involvement in SARS-CoV-2 infection: a review of the current pediatric literature. Front Neurol. (2023) 14:1134507. doi: 10.3389/fneur.2023.1134507 37305745 PMC10249431

[131] Szewczyk AK Skrobas U Jamroz-Wiśniewska A Mitosek-Szewczyk K Rejdak K. Facial diplegia—Complication or manifestation of SARS-coV-2 infection? A case report and systemic literature review. Healthc (Basel). (2021) 9(11):1492. doi: 10.3390/healthcare9111492 PMC8618007 34828542

[132] Vagge A Giannaccare G Scarinci F Cacciamani A Pellegrini M Bernabei F. Acute acquired concomitant esotropia from excessive application of near vision during the COVID-19 lockdown. J Pediatr Ophthalmol Strabismus. (2021) 57:e88–91. doi: 10.3928/01913913-20200828-01 33090234

[133] Mohan A Sen P Mujumdar D Shah C Jain E. Series of cases of acute acquired comitant esotropia in children associated with excessive online classes on smartphone during COVID-19 pandemic; digital eye strain among kids (DESK) study-3. Strabismus. (2021) 29(3):163–7. doi: 10.1080/09273972.2021.1948072 34223812

[134] Sanguinetti SY Ramdhani RA. Opsoclonus-myoclonus-ataxia syndrome related to the novel coronavirus (COVID-19). J Neuroophthal. (2021) 41(3):e288–9. doi: 10.1097/WNO.0000000000001129 PMC8366529 32925477

[135] Pereira LA Soares LCM Nascimento PA Cirillo LRN Sakuma HT Veiga GLD. Retinal findings in hospitalised patients with severe COVID-19. Br J Ophthalmol. (2022) 106(1):102–5. doi: 10.1136/bjophthalmol-2020-317576 33067361

[136] Zhang X Chen X Chen L Deng C Zou X Liu W. The evidence of SARS-CoV-2 infection on ocular surface. Ocular Surface. (2020) 18(3):360–2. doi: 10.1016/j.jtos.2020.03.010 PMC7194535 32289466

[137] Loffredo L Pacella F Pacella E Tiscione G Oliva A Violi F. Conjunctivitis and COVID-19: A meta-analysis. J Med Virol. (2020) 92(9):1413–4. doi: 10.1002/jmv.25938 PMC7264785 32330304

[138] Chen L Deng C Chen X Zhang X Chen B Yu H. Ocular manifestations and clinical characteristics of 535 cases of COVID-19 in Wuhan, China: a cross-sectional study. Acta Ophthalmol. (2020) 98(8):e951–9. doi: 10.1111/aos.14472 PMC7276826 32421258

[139] Guan Wj Ni Z Hu Y Liang WH Ou CQ He JX. Clinical characteristics of coronavirus disease 2019 in China. N Engl J Med. (2020) 382(18):NEJMoa2002032. doi: 10.1056/NEJMoa2002032 32109013 PMC7092819

[140] Xia J Tong J Liu M Shen Y Guo D. Evaluation of coronavirus in tears and conjunctival secretions of patients with SARS-CoV-2 infection. J Med Virol. (2020) 92(6):589–94. doi: 10.1002/jmv.25725 PMC7228294 32100876

[141] Bourdon H Jaillant R Ballino A El Kaim P Debillon L Bodin S. Teleconsultation in primary ophthalmic emergencies during the COVID-19 lockdown in Paris: Experience with 500 patients in March and April 2020. J Français d’Ophtalmol. (2020) 43(7):577–85. doi: 10.1016/j.jfo.2020.05.005 PMC7284250 32564983

[142] Rokohl AC Loreck N Wawer Matos PA Zwingelberg S Augustin M Dewald F. More than loss of taste and smell: burning watering eyes in coronavirus disease 2019. Clin Microbiol Infect. (2020) 26(11):1560.e5–1560.e8. doi: 10.1016/j.cmi.2020.08.018 PMC7442009 32835793

[143] Sindhuja K Lomi N Asif MI Tandon R. Clinical profile and prevalence of conjunctivitis in mild COVID-19 patients in a tertiary care COVID-19 hospital: A retrospective cross-sectional study. Indian J Ophthalmol. (2020) 68(8):1546–50. doi: 10.4103/ijo.IJO_1319_20 PMC7640841 32709772

[144] Abrishami M Tohidinezhad F Daneshvar R Omidtabrizi A Amini M Sedaghat A. Ocular manifestations of hospitalized patients with COVID-19 in northeast of Iran. Ocular Immunol Inflammation. (2020) 28(5):739–44. doi: 10.1080/09273948.2020.1773868 32569494

[145] Valente P Iarossi G Federici M Petroni S Palma P Cotugno N. Ocular manifestations and viral shedding in tears of pediatric patients with coronavirus disease 2019: a preliminary report. J Am Assoc Pediatr Ophthalmol Strabismus. (2020) 24(4):212–5. doi: 10.1016/j.jaapos.2020.05.002 PMC7282793 32531341

[146] Meduri A Oliverio GW Mancuso G Giuffrida A Guarneri C Venanzi Rullo E. Ocular surface manifestation of COVID-19 and tear film analysis. Sci Rep. (2020) 10(1):20178. doi: 10.1038/s41598-020-77194-9 33214658 PMC7677531

[147] Invernizzi A Torre A Parrulli S Zicarelli F Schiuma M Colombo V. Retinal findings in patients with COVID-19: Results from the SERPICO-19 study. EClinicalMedicine. (2020) 27:100550. doi: 10.1016/j.eclinm.2020.100550 32984785 PMC7502280

[148] Pirraglia MP Ceccarelli G Cerini A Visioli G d'Ettorre G Mastroianni CM. Retinal involvement and ocular findings in COVID-19 pneumonia patients. Sci Rep. (2020) 10(1):17419. doi: 10.1038/s41598-020-74446-6 33060700 PMC7566835

[149] Lee YH Kim YC Shin JP. Characteristics of ocular manifestations of patients with coronavirus disease 2019 in daegu province, korea. J Korean Med Sci. (2020) 35(35):e322. doi: 10.13048/jkm.20024 32893523 PMC7476796

[150] Landecho MF Yuste JR Gándara E Sunsundegui P Quiroga J Alcaide AB. COVID-19 retinal microangiopathy as an *in vivo* biomarker of systemic vascular disease? J Intern Med. (2021) 289(1):116–20. doi: 10.1111/joim.13156 32729633

[151] Öncül H Öncül FY Alakus MF Çağlayan M Dag U. Ocular findings in patients with coronavirus disease 2019 (COVID-19) in an outbreak hospital. J Med Virol. (2021) 93(2):1126–32. doi: 10.1002/jmv.26412 PMC7436579 32776614

[152] Ng XL Betzler BK Testi I Ho SL Tien M Ngo WK. Ocular adverse events after COVID-19 vaccination. Ocul Immunol Inflammation. 29(6):1216–24. doi: 10.1080/09273948.2021.1976221 PMC8477588 34559576

[153] Ferrand N Accorinti M Agarwal M Spartalis C Manni P Stuebiger N. COVID-19 vaccination and uveitis: epidemiology, clinical features and visual prognosis. Ocular Immunol Inflammation. (2022) 30(5):1265–73. doi: 10.1080/09273948.2022.2058964 35404757

[154] Zhong Z Wu Q Lai Y Dai L Gao Y Liao W. Risk for uveitis relapse after COVID-19 vaccination. J Autoimmun. (2022) 133:102925. doi: 10.1016/j.jaut.2022.102925 36209692 PMC9531657

[155] Singh RB Parmar UPS Kahale F Agarwal A Tsui E. Vaccine-associated uveitis after COVID-19 vaccination. Ophthalmology. (2023) 130(2):179–86. doi: 10.1016/j.ophtha.2022.08.027 PMC9428109 36055601

[156] García-Estrada C Gómez-Figueroa E Alban L Arias-Cárdenas A. Optic neuritis after COVID-19 vaccine application. Clin Exp Neuroimmunol. (2022) 13(2):72–4. doi: 10.1111/cen3.12682 PMC8653244 34900001

[157] Lee WJA. COVID-19 vaccine associated optic neuritis. QJM. (2022) 115(10):683–5. doi: 10.1093/qjmed/hcac208 PMC9452137 36040199

[158] Katayama H Itoh K Hashimoto M. Bilateral Optic Neuritis after COVID-19 mRNA Vaccination. Case Rep Ophthalmol. (2022) 13(2):578–83. doi: 10.1159/000525938 PMC9459513 36160483

[159] Sawalha K Adeodokun S Kamoga GR. COVID-19-induced acute bilateral optic neuritis. J Invest Med High Impact Case Rep. (2020) 8:2324709620976018. doi: 10.1177/2324709620976018 PMC7705770 33238757

[160] Yeo S Kim H Lee J Yi J Chung YR. Retinal vascular occlusions in COVID-19 infection and vaccination: a literature review. Graefes Arch Clin Exp Ophthalmol. (2023) 261(7):1793–808. doi: 10.1007/s00417-022-05953-7 PMC9811047 36598554

[161] Montano D. Frequency and associations of adverse reactions of COVID-19 vaccines reported to pharmacovigilance systems in the European Union and the United States. Front Public Health. (2022) 9:756633. doi: 10.3389/fpubh.2021.756633 35186864 PMC8850379

[162] Gronbeck C Grzybowski A Grant-Kels JM. COVID-19 and the eye. Clinics Dermatol. (2024) 42(1):17–24. doi: 10.1016/j.clindermatol.2023.10.008 37865278

[163] Zhang P Wu Q Xu X Chen M. A case of IgG4-related ophthalmic disease after SARS-CoV-2 vaccination: case report and literature review. Front Immunol. (2024) 15:1303589. doi: 10.3389/fimmu.2024.1303589 38455056 PMC10917890

[164] Muller I Consonni D Crivicich E Di Marco F Currò N Salvi M. Increased risk of thyroid eye disease following covid-19 vaccination. J Clin Endocrinol Metab. (2023) 109(2):516–26. doi: 10.1210/clinem/dgad501 PMC10795895 37622279

[165] Moon Y Jung JH Shin HJ Choi DG Park KA Jeon H. Non-arteritic ischemic optic neuropathy following COVID-19 vaccination in Korea: A case series. J Korean Med Sci. (2023) 38(12):e95. doi: 10.3346/jkms.2023.38.e95 36974402 PMC10042731

[166] Karagöz IK Munk MR Kaya M Rückert R Yıldırım M Karabaş L. Using bioinformatic protein sequence similarity to investigate if SARS CoV-2 infection could cause an ocular autoimmune inflammatory reactions? Exp Eye Res. (2021) 203:108433. doi: 10.1016/j.exer.2020.108433 33400927 PMC7831665

[167] Monje M Iwasaki A. The neurobiology of long COVID. Neuron. (2022) 110(21):3484–96. doi: 10.1016/j.neuron.2022.10.006 PMC9537254 36288726

[168] Ashkenazy N Patel NA Sridhar J Yannuzzi NA Belin PJ Kaplan R. Hemi- and central retinal vein occlusion associated with COVID-19 infection in young patients without known risk factors. Ophthalmol Retina. (2022) 6(6):520–30. doi: 10.1016/j.oret.2022.02.004 PMC8907133 35278727

[169] Huang Y Chen T Chen X Wan L Hou X Zhuang J. Corneal stroma analysis and related ocular manifestations in recovered COVID-19 patients. Invest Ophthalmol Vis Sci. (2024) 65(5):14. doi: 10.1167/iovs.65.5.14 PMC11086707 38713483

[170] Gao Y Zhang Y Mou K Liu Y Chen Q Man S. Assessment of alterations in the retina and vitreous in pre- and post-COVID-19 patients using swept-source optical coherence tomography and angiography: A comparative study. J Med Virol. (2023) 95(10):e29168. doi: 10.1002/jmv.v95.10 37815403

[171] Srichawla BS Kipkorir V Manan MR Dhali A Diebel S Sawant T. Stealth invaders: unraveling the mystery of neurotropic viruses and their elusive presence in cerebrospinal fluid – a comprehensive review. Ann Med Surg (Lond). (2023) 85(6):2761–6. doi: 10.1097/MS9.0000000000000736 PMC10289609 37363567

[172] Pellett PE Mitra S Holland TC. Basics of virology. Handb Clin Neurol. (2014) 123:45–66. doi: 10.1016/B978-0-444-53488-0.00002-X 25015480 PMC7152233

[173] Ruiz-Pablos M Paiva B Montero-Mateo R Garcia N Zabaleta A. Epstein-barr virus and the origin of myalgic encephalomyelitis or chronic fatigue syndrome. Front Immunol. (2021) 12:656797. doi: 10.3389/fimmu.2021.656797 34867935 PMC8634673

[174] Su Y Yuan D Chen DG Ng RH Wang K Choi J. Multiple early factors anticipate post-acute COVID-19 sequelae. Cell. (2022) 185(5):881–895.e20. doi: 10.1016/j.cell.2022.01.014 35216672 PMC8786632

[175] Chin MS Hooper LC Hooks JJ Detrick B. Identification of α-fodrin as an autoantigen in experimental coronavirus retinopathy (ECOR). J Neuroimmunol. (2014) 272(1-2):42–50. doi: 10.1016/j.jneuroim.2014.05.002 24864013 PMC7112846

[176] Vinores SA Wang Y Vinores MA Derevjanik NL Shi A Klein DA. Blood–retinal barrier breakdown in experimental coronavirus retinopathy: association with viral antigen, inflammation, and VEGF in sensitive and resistant strains. J Neuroimmunol. (2001) 119(2):175–82. doi: 10.1016/S0165-5728(01)00374-5 PMC7119735 11585619

[177] Wang Y Detrick B Yu ZX Zhang J Chesky L Hooks JJ. The role of apoptosis within the retina of coronavirus-infected mice. Invest Ophthalmol Visual Sci. (2000) 41(10):3011–8. 10967058

[178] Babu K Konana VK Ganesh SK Patnaik G Chan NSW Chee SP. Viral anterior uveitis. Indian J Ophthalmol. (2020) 68(9):1764–73. doi: 10.4103/ijo.IJO_928_20 PMC7690545 32823392

[179] Lulla V Sridhar A. Understanding neurotropic enteric viruses: routes of infection and mechanisms of attenuation. Cell Mol Life Sci. (2024) 81(1):413. doi: 10.1007/s00018-024-05450-6 39365457 PMC11452578

[180] Murira A Lamarre A. Type-I interferon responses: from friend to foe in the battle against chronic viral infection. Front Immunol. (2016) 7:609. doi: 10.3389/fimmu.2016.00609 28066419 PMC5165262

[181] de Oliveira Souza R Duarte Júnior JWB Della Casa VS Santoro Rosa D Renia L Claser C. Unraveling the complex interplay: immunopathology and immune evasion strategies of alphaviruses with emphasis on neurological implications. Front Cell Infect Microbiol. (2024) 14:1421571. doi: 10.3389/fcimb.2024.1421571 39211797 PMC11358129

[182] Al-Obaidi MMJ Bahadoran A Wang SM Manikam R Raju CS Sekaran SD. Disruption of the blood brain barrier is vital property of neurotropic viral infection of the central nervous system. av. (2018) 62(1):16–27. doi: 10.4149/av_2018_102 29521099

[183] Qiao H Deng X Qiu L Qu Y Chiu Y Chen F. SARS-CoV-2 induces blood-brain barrier and choroid plexus barrier impairments and vascular inflammation in mice. J Med Virol. (2024) 96(5):e29671. doi: 10.1002/jmv.29671 38747003 PMC11446308

[184] Paterson RW Brown RL Benjamin L Nortley R Wiethoff S Bharucha T. The emerging spectrum of COVID-19 neurology: clinical, radiological and laboratory findings. Brain. (2020) 143(10):3104–20. doi: 10.1093/brain/awaa240 PMC7454352 32637987

[185] Ryan FJ Hope CM Masavuli MG Lynn MA Mekonnen ZA Yeow AEL. Long-term perturbation of the peripheral immune system months after SARS-CoV-2 infection. BMC Med. (2022) 20(1):26. doi: 10.1186/s12916-021-02228-6 35027067 PMC8758383

[186] Heming M Li X Räuber S Mausberg AK Börsch AL Hartlehnert M. Neurological manifestations of COVID-19 feature T cell exhaustion and dedifferentiated monocytes in cerebrospinal fluid. Immunity. (2021) 54(1):164–175.e6. doi: 10.1016/j.immuni.2020.12.011 33382973 PMC7831653

[187] Ansari A Arya R Sachan S Jha SN Kalia A Lall A. Immune memory in mild COVID-19 patients and unexposed donors reveals persistent T cell responses after SARS-coV-2 infection. Front Immunol. (2021) 12:636768. doi: 10.3389/fimmu.2021.636768 33777028 PMC7991090

[188] Pellegrini L Albecka A Mallery DL Kellner MJ Paul D Carter AP. SARS-coV-2 infects the brain choroid plexus and disrupts the blood-CSF barrier in human brain organoids. Cell Stem Cell. (2020) 27(6):951–961.e5. doi: 10.1016/j.stem.2020.10.001 33113348 PMC7553118

[189] Neumann B Schmidbauer ML Dimitriadis K Otto S Knier B Niesen WD. Cerebrospinal fluid findings in COVID-19 patients with neurological symptoms. J Neurol Sci. (2020) 418:117090. doi: 10.1016/j.jns.2020.117090 32805440 PMC7417278

[190] Lui KO Ma Z Dimmeler S. SARS-CoV-2 induced vascular endothelial dysfunction: direct or indirect effects? Cardiovasc Res. (2024) 120(1):34–43. doi: 10.1093/cvr/cvad191 38159046

[191] Wang P Jin L Zhang M Wu Y Duan Z Guo Y. Blood–brain barrier injury and neuroinflammation induced by SARS-CoV-2 in a lung–brain microphysiological system. Nat BioMed Eng. (2024) 8(8):1053–68. doi: 10.1038/s41551-023-01054-w 37349391

[192] Prieto-Villalobos J Lucero CM Rovegno M Gómez GI Retamal MA Orellana JA. SARS-CoV-2 spike protein S1 activates Cx43 hemichannels and disturbs intracellular Ca2+ dynamics. Biol Res. (2023) 56(1):56. doi: 10.1186/s40659-023-00468-9 37876016 PMC10598968

[193] Mehta P McAuley DF Brown M Sanchez E Tattersall RS Manson JJ. COVID-19: consider cytokine storm syndromes and immunosuppression. Lancet. (2020) 395(10229):1033–4. doi: 10.1016/S0140-6736(20)30628-0 PMC7270045 32192578

[194] Tay MZ Poh CM Rénia L MacAry PA Ng LFP. The trinity of COVID-19: immunity, inflammation and intervention. Nat Rev Immunol. (2020) 20(6):363–74. doi: 10.1038/s41577-020-0311-8 PMC7187672 32346093

[195] Huang C Wang Y Li X Ren L Zhao J Hu Y. Clinical features of patients infected with 2019 novel coronavirus in Wuhan, China. Lancet. (2020) 395(10223):497–506. doi: 10.1016/S0140-6736(20)30183-5 31986264 PMC7159299

[196] Chen G Wu D Guo W Cao Y Huang D Wang H. Clinical and immunological features of severe and moderate coronavirus disease 2019. J Clin Invest. (2020) 130(5):2620–9. doi: 10.1172/JCI137244 PMC7190990 32217835

[197] Ahmad I Rathore FA. Neurological manifestations and complications of COVID-19: A literature review. J Clin Neurosci. (2020) 77:8–12. doi: 10.1016/j.jocn.2020.05.017 32409215 PMC7200361

[198] Song E Zhang C Israelow B Lu-Culligan A Prado AV Skriabine S. Neuroinvasion of SARS-CoV-2 in human and mouse brain. J Exp Med. (2021) 218(3):e20202135. doi: 10.1084/jem.20202135 33433624 PMC7808299

[199] Ghasemi M Umeton RP Keyhanian K Mohit B Rahimian N Eshaghhosseiny N. SARS-coV-2 and acute cerebrovascular events: an overview. J Clin Med. (2021) 10(15):3349. doi: 10.3390/jcm10153349 34362133 PMC8348889

[200] Etter MM Martins TA Kulsvehagen L Pössnecker E Duchemin W Hogan S. Severe Neuro-COVID is associated with peripheral immune signatures, autoimmunity and neurodegeneration: a prospective cross-sectional study. Nat Commun. (2022) 13(1):6777. doi: 10.1038/s41467-022-34068-0 36351919 PMC9645766

[201] Buzhdygan TP DeOre BJ Baldwin-Leclair A Buzhdygan TP DeOre BJ Baldwin-Leclair A. The SARS-CoV-2 spike protein alters barrier function in 2D static and 3D microfluidic *in-vitro* models of the human blood–brain barrier. Neurobiol Dis. (2020) 146:105131. doi: 10.1016/j.nbd.2020.105131 33053430 PMC7547916

[202] Yang X Yu Y Xu J Shu H Xia J Liu H. Clinical course and outcomes of critically ill patients with SARS-CoV-2 pneumonia in Wuhan, China: a single-centered, retrospective, observational study. Lancet Respir Med. (2020) 8(5):475–81. doi: 10.1016/S2213-2600(20)30079-5 PMC7102538 32105632

[203] Mingoti MED Bertollo AG Simões JLB Francisco GR Bagatini MD Ignácio ZM. COVID-19, oxidative stress, and neuroinflammation in the depression route. J Mol Neurosci. (2022) 72(6):1166–81. doi: 10.1007/s12031-022-02004-y PMC8942178 35322375

[204] Yi JS Cox MA Zajac AJ. T-cell exhaustion: characteristics, causes and conversion. Immunology. (2010) 129(4):474–81. doi: 10.1111/j.1365-2567.2010.03255.x PMC2842494 20201977

[205] Schwabenland M Salié H Tanevski J Killmer S Lago MS Schlaak AE. Deep spatial profiling of human COVID-19 brains reveals neuroinflammation with distinct microanatomical microglia-T-cell interactions. Immunity. (2021) 54(7):1594–1610.e11. doi: 10.1016/j.immuni.2021.06.002 34174183 PMC8188302

[206] Li Y Fu L Gonzales DM Lavi E. Coronavirus neurovirulence correlates with the ability of the virus to induce proinflammatory cytokine signals from astrocytes and microglia. J Virol. (2004) 78(7):3398–406. doi: 10.1128/JVI.78.7.3398-3406.2004 PMC371061 15016862

[207] Townsend L Dyer AH Naughton A Kiersey R Holden D Gardiner M. Longitudinal analysis of COVID-19 patients shows age-associated T cell changes independent of ongoing ill-health. Front Immunol. (2021) 12:676932. doi: 10.3389/fimmu.2021.676932 34025675 PMC8138306

[208] Yin Y Cui Q Li Y Irwin N Fischer D Harvey AR. Macrophage-derived factors stimulate optic nerve regeneration. J Neurosci. (2003) 23(6):2284–93. doi: 10.1523/JNEUROSCI.23-06-02284.2003 PMC6742044 12657687

[209] Tanaka Y Adams DH Hubscher S Hirano H Siebenlist U Shaw S. T-cell adhesion induced by proteoglycan-immobilized cytokine MIP-1 beta. Nature. (1993) 361(6407):79–82. doi: 10.1038/361079a0 7678446

[210] Peng Y Mentzer AJ Liu G Yao X Yin Z Dong D. Broad and strong memory CD4+ and CD8+ T cells induced by SARS-CoV-2 in UK convalescent individuals following COVID-19. Nat Immunol. (2020) 21(11):1336–45. doi: 10.1038/s41590-020-0782-6 PMC7611020 32887977

[211] Yi Y Lagniton PNP Ye S Li E Xu RH. COVID-19: what has been learned and to be learned about the novel coronavirus disease. Int J Biol Sci. (2020) 16(10):1753–66. doi: 10.7150/ijbs.45134 PMC7098028 32226295

[212] Azkur AK Akdis M Azkur D Sokolowska M van de Veen W Brüggen MC. Immune response to SARS-CoV-2 and mechanisms of immunopathological changes in COVID-19. Allergy. (2020) 75(7):1564–81. doi: 10.1111/all.14364 PMC7272948 32396996

[213] Pelaia C Tinello C Vatrella A De Sarro G Pelaia G. Lung under attack by COVID-19-induced cytokine storm: pathogenic mechanisms and therapeutic implications. Ther Adv Respir Dis. (2020) 14:1753466620933508. doi: 10.1177/1753466620933508 32539627 PMC7298425

[214] Kim JS Lee JY Yang JW Lee KH Effenberger M Szpirt W. Immunopathogenesis and treatment of cytokine storm in COVID-19. Theranostics. (2021) 11(1):316–29. doi: 10.7150/thno.49713 PMC7681075 33391477

[215] Vibholm LK Nielsen SS Pahus MH Frattari GS Olesen R Andersen R. SARS-CoV-2 persistence is associated with antigen-specific CD8 T-cell responses. EBioMedicine. (2021) 64:103230. doi: 10.1016/j.ebiom.2021.103230 33530000 PMC7847186

[216] Peluso MJ Deitchman AN Torres L Iyer NS Munter SE Nixon CC. Long-term SARS-CoV-2-specific immune and inflammatory responses in individuals recovering from COVID-19 with and without post-acute symptoms. Cell Rep. (2021) 36(6):109518. doi: 10.1016/j.celrep.2021.109518 34358460 PMC8342976

[217] Verkhratsky A Parpura V. Astrogliopathology in neurological, neurodevelopmental and psychiatric disorders. Neurobiol Dis. (2016) 85:254–61. doi: 10.1016/j.nbd.2015.03.025 PMC4592688 25843667

[218] Wenzel J Lampe J Müller-Fielitz H Schuster R Zille M Müller K. The SARS-CoV-2 main protease Mpro causes microvascular brain pathology by cleaving NEMO in brain endothelial cells. Nat Neurosci. (2021) 24(11):1522–33. doi: 10.1038/s41593-021-00926-1 PMC8553622 34675436

[219] Samudyata Oliveira AO Malwade S Rufino de Sousa N Goparaju SK Gracias J. SARS-CoV-2 promotes microglial synapse elimination in human brain organoids. Mol Psychiatry. (2022) 27(10):3939–50. doi: 10.1038/s41380-022-01786-2 PMC9533278 36198765

[220] Thakur KT Miller EH Glendinning MD Al-Dalahmah O Banu MA Boehme AK. COVID-19 neuropathology at Columbia University Irving Medical Center/New York Presbyterian Hospital. Brain. (2021) 144(9):2696. doi: 10.1093/brain/awab148 33856027 PMC8083258

[221] Delgado-Roche L Mesta F. Oxidative stress as key player in severe acute respiratory syndrome coronavirus (SARS-CoV) infection. Arch Med Res. (2020) 51(5):384–7. doi: 10.1016/j.arcmed.2020.04.019 PMC7190501 32402576

[222] Forcados GE Muhammad A Oladipo OO Makama S Meseko CA. Metabolic implications of oxidative stress and inflammatory process in SARS-coV-2 pathogenesis: therapeutic potential of natural antioxidants. Front Cell Infect Microbiol. (2021) 11:654813. doi: 10.3389/fcimb.2021.654813 34123871 PMC8188981

[223] Ernst T Ryan MC Liang HJ Wang JP Cunningham E Saleh MG. Neuronal and glial metabolite abnormalities in participants with persistent neuropsychiatric symptoms after COVID-19: A brain proton magnetic resonance spectroscopy study. J Infect Dis. (2023) 228(11):1559–70. doi: 10.1093/infdis/jiad309 PMC10681871 37540098

[224] Sandeep Subba R Mondal AC. Does COVID-19 trigger the risk for the development of Parkinson’s disease? Therapeutic potential of vitamin C. Mol Neurobiol. (2023) 61(12):9945–60. doi: 10.1007/s12035-023-03756-3 37957424

[225] Chaudhry ZL Klenja D Janjua N Cami-Kobeci G Ahmed BY. COVID-19 and Parkinson’s disease: shared inflammatory pathways under oxidative stress. Brain Sci. (2020) 10(11):807. doi: 10.3390/brainsci10110807 33142819 PMC7693814

[226] Taquet M Geddes JR Husain M Luciano S Harrison PJ. 6-month neurological and psychiatric outcomes in 236 379 survivors of COVID-19: a retrospective cohort study using electronic health records. Lancet Psychiatry. (2021) 8(5):416–27. doi: 10.1016/S2215-0366(21)00084-5 PMC8023694 33836148

[227] Estiri H Strasser ZH Brat GA Semenov YR Consortium for Characterization of COVID-19 by EHR (4CE) Patel CJ. Evolving phenotypes of non-hospitalized patients that indicate long COVID. BMC Med. (2021) 19(1):249. doi: 10.1186/s12916-021-02115-0 34565368 PMC8474909

[228] Premraj L Kannapadi NV Briggs J Seal SM Battaglini D Fanning J. Mid and long-term neurological and neuropsychiatric manifestations of post-COVID-19 syndrome: A meta-analysis. J Neurol Sci. (2022) 434(9):120162. doi: 10.1016/j.jns.2022.120162 35121209 PMC8798975

[229] Chang SE Feng A Meng W Apostolidis SA Mack E Artandi M. New-onset IgG autoantibodies in hospitalized patients with COVID-19. Nat Commun. (2021) 12(1):5417. doi: 10.1038/s41467-021-25509-3 34521836 PMC8440763

[230] Arthur JM Forrest JC Boehme KW Kennedy JL Owens S Herzog C. Development of ACE2 autoantibodies after SARS-CoV-2 infection. PloS One. (2021) 16(9):e0257016. doi: 10.1371/journal.pone.0257016 34478478 PMC8415618

[231] Sacchi MC Tamiazzo S Stobbione P Agatea L De Gaspari P Stecca A. SARS-CoV-2 infection as a trigger of autoimmune response. Clin Transl Sci. (2021) 14(3):898–907. doi: 10.1111/cts.12953 33306235 PMC8212749

[232] Peluso MJ Deeks SG. Early clues regarding the pathogenesis of long-COVID. Trends Immunol. (2022) 43(4):268–70. doi: 10.1016/j.it.2022.02.008 PMC8901423 35272932

[233] Wallukat G Hohberger B Wenzel K Fürst J Schulze-Rothe S Wallukat A. Functional autoantibodies against G-protein coupled receptors in patients with persistent Long-COVID-19 symptoms. J Transl Autoimmun. (2021) 4:100100. doi: 10.1016/j.jtauto.2021.100100 33880442 PMC8049853

[234] Rieder GS Nogara PA Omage FB Duarte T Dalla Corte CL da Rocha JBT. Computational analysis of the interactions between Ebselen and derivatives with the active site of the main protease from SARS-CoV-2. Comput Biol Chem. (2023) 107:107956. doi: 10.1016/j.compbiolchem.2023.107956 37748316

[235] Hammond J Leister-Tebbe H Gardner A Abreu P Bao W Wisemandle W. Oral nirmatrelvir for high-risk, nonhospitalized adults with covid-19. N Engl J Med. (2022) 386(15):1397–408. doi: 10.1056/NEJMoa2118542 PMC8908851 35172054

[236] Wen W Chen C Tang J Wang C Zhou M Cheng Y. Efficacy and safety of three new oral antiviral treatment (molnupiravir, fluvoxamine and Paxlovid) for COVID-19:a meta-analysis. Ann Med. 54(1):516–23. doi: 10.1080/07853890.2022.2034936 PMC8820829 35118917

[237] Esposito G Pesce M Seguella L Sanseverino W Lu J Corpetti C. The potential of cannabidiol in the COVID-19 pandemic. Br J Pharmacol. (2020) 177(21):4967–70. doi: 10.1111/bph.v177.21 PMC7300643 32519753

[238] Scarante FF Ribeiro MA Almeida-Santos AF Guimarães FS Campos AC. Glial cells and their contribution to the mechanisms of action of cannabidiol in neuropsychiatric disorders. Front Pharmacol. (2021) 11:618065. doi: 10.3389/fphar.2020.618065 33613284 PMC7890128

[239] Campos AC Fogaça MV Sonego AB Guimarães FS. Cannabidiol, neuroprotection and neuropsychiatric disorders. Pharmacol Res. (2016) 112:119–27. doi: 10.1016/j.phrs.2016.01.033 26845349

[240] Khan FI Kang T Ali H Lai D. Remdesivir strongly binds to RNA-dependent RNA polymerase, membrane protein, and main protease of SARS-CoV-2: indication from molecular modeling and simulations. Front Pharmacol. (2021) 12:710778. doi: 10.3389/fphar.2021.710778 34305617 PMC8293383

[241] Bernal E García-Villalba E Pons E Hernández MD Báguena C Puche G. Remdesivir plus dexamethasone is associated to improve the clinical outcome of COVID-19 hospitalized patients regardless of their vaccination status. Med Clin (Barc). (2023) 161(4):139–46. doi: 10.1016/j.medcle.2023.03.027 PMC10073576 37100681

[242] Putilina MV Grishin DV. SARS-CoV-2 (COVID-19) as a predictor of neuroinflammation and neurodegeneration: potential therapy strategies. Zh Nevrol Psikhiatr Im S S Korsakova. (2020) 120(8. Vyp. 2):58–64. doi: 10.17116/jnevro202012008258 33016678

[243] Suran M. VA finds nirmatrelvir associated with lower risk of long COVID. JAMA. (2022) 328(24):2386. doi: 10.1001/jama.2022.20051 36573990

[244] Peluso MJ Anglin K Durstenfeld MS Martin JN Kelly JD Hsue PY. Effect of oral nirmatrelvir on long COVID symptoms: 4 cases and rationale for systematic studies. Pathog Immun. (2022) 7(1):95–103. doi: 10.20411/pai.v7i1.518 35800257 PMC9254867

[245] Hung YP Lee JC Chiu CW Lee CC Tsai PJ Hsu IL. Oral nirmatrelvir/ritonavir therapy for COVID-19: the dawn in the dark? Antibiot (Basel). (2022) 11(2):220. doi: 10.3390/antibiotics11020220 PMC8868411 35203821

[246] Visvabharathy L Orban ZS Koralnik IJ. Case report: Treatment of long COVID with a SARS-CoV-2 antiviral and IL-6 blockade in a patient with rheumatoid arthritis and SARS-CoV-2 antigen persistence. Front Med (Lausanne). (2022) 9:1003103. doi: 10.3389/fmed.2022.1003103 36213654 PMC9537824

[247] Ayoubkhani D Bermingham C Pouwels KB Glickman M Nafilyan V Zaccardi F. Trajectory of long covid symptoms after covid-19 vaccination: community based cohort study. BMJ. (2022) 377:e069676. doi: 10.1136/bmj-2021-069676 35584816 PMC9115603

[248] Chaves Filho AJM Gonçalves F Mottin M Andrade CH Fonseca SNS Macedo DS. Repurposing of tetracyclines for COVID-19 neurological and neuropsychiatric manifestations: A valid option to control SARS-coV-2-associated neuroinflammation? J Neuroimmune Pharmacol. (2021) 16(2):213–8. doi: 10.1007/s11481-021-09986-3 PMC7854870 33534108

[249] Pelaia C Calabrese C Garofalo E Bruni A Vatrella A Pelaia G. Therapeutic role of tocilizumab in SARS-coV-2-induced cytokine storm: rationale and current evidence. Int J Mol Sci. (2021) 22(6):3059. doi: 10.3390/ijms22063059 33802761 PMC8002419

[250] Zhang X Zhang Y Qiao W Zhang J Qi Z. Baricitinib, a drug with potential effect to prevent SARS-COV-2 from entering target cells and control cytokine storm induced by COVID-19. Int Immunopharmacol. (2020) 86:106749. doi: 10.1016/j.intimp.2020.106749 32645632 PMC7328558

[251] Shih LJ Yang CC Liao MT Lu KC Hu WC Lin CP. An important call: Suggestion of using IL-10 as therapeutic agent for COVID-19 with ARDS and other complications. Virulence. 14(1):2190650. doi: 10.1080/21505594.2023.2190650 PMC10026935 36914565

[252] Spuch C López-García M Rivera-Baltanás T Cabrera-Alvargonzález JJ Gadh S Rodrigues-Amorim D. Efficacy and safety of lithium treatment in SARS-coV-2 infected patients. Front Pharmacol. (2022) 13:850583. doi: 10.3389/fphar.2022.850583 35496309 PMC9046673

[253] Liu TH Ho CH Chen DTL Wu JY Huang PY Lai CC. Omega-3 polyunsaturated fatty acids and the psychiatric post-acute sequelae of COVID-19: A one-year retrospective cohort analysis of 33,908 patients. Brain Behavior Immun. (2023) 114:453–61. doi: 10.1016/j.bbi.2023.09.008 37716377

[254] Zaa CA Espitia C Reyes-Barrera KL An Z Velasco-Velázquez MA. Neuroprotective agents with therapeutic potential for COVID-19. Biomolecules. (2023) 13(11):1585. doi: 10.3390/biom13111585 38002267 PMC10669388

[255] Di Pierro F Iqtadar S Khan A Ullah Mumtaz S Masud Chaudhry M Bertuccioli A. Potential clinical benefits of quercetin in the early stage of COVID-19: results of a second, pilot, randomized, controlled and open-label clinical trial. Int J Gen Med. (2021) 14:2807–16. doi: 10.2147/IJGM.S318949 PMC8238537 34194240

[256] Hassaniazad M Eftekhar E Inchehsablagh BR Kamali H Tousi A Jaafari MR. A triple-blind, placebo-controlled, randomized clinical trial to evaluate the effect of curcumin-containing nanomicelles on cellular immune responses subtypes and clinical outcome in COVID-19 patients. Phytother Res. (2021) 35(11):6417–27. doi: 10.1002/ptr.v35.11 PMC8661812 34541720

[257] Vollbracht C Kraft K. Feasibility of vitamin C in the treatment of post viral fatigue with focus on long COVID, based on a systematic review of IV vitamin C on fatigue. Nutrients. (2021) 13(4):1154. doi: 10.3390/nu13041154 33807280 PMC8066596

[258] Drucker E Krapfenbauer K. Pitfalls and limitations in translation from biomarker discovery to clinical utility in predictive and personalised medicine. EPMA J. (2013) 4(1):7. doi: 10.1186/1878-5085-4-7 23442211 PMC3599714

[259] Racette L Abu SL Poleon S Thomas T Sabbagh N Girkin CA. The impact of the coronavirus disease 2019 pandemic on adherence to ocular hypotensive medication in patients with primary open-angle glaucoma. Ophthalmology. (2022) 129(3):258–66. doi: 10.1016/j.ophtha.2021.10.009 PMC8523310 34673098

